# Chronic dehydration induces injury pathways in rats, but does not mimic histopathology of chronic interstitial nephritis in agricultural communities

**DOI:** 10.1038/s41598-023-43567-z

**Published:** 2023-10-23

**Authors:** Gerd Schreurs, Stuart Maudsley, Cynthia Nast, Marleen Praet, Sylvina Da Silva Fernandes, Peter Boor, Patrick D’Haese, Marc E. De Broe, Benjamin A. Vervaet

**Affiliations:** 1 Laboratory of Pathophysiology, University of Antwerp, Universiteitsplein 1, 2610 Antwerp, Belgium; 2 Receptor Biology Lab, Department of Biomedical Science, University of Antwerp, Antwerp, Belgium; 3 Cedars-Sinai Medical Center, Los Angeles, USA; 4 Department of Pathology, Ghent University Hospital, Ghent, Belgium; 5 Institute of Pathology, Electron Microscopy Facility and Division of Nephrology and Immunology, RWTH Aachen University Hospital, Aachen, Germany; 6 Institute of Pathology, RWTH Aachen University Hospital, Aachen, Germany

**Keywords:** Nephrology, Kidney diseases, Experimental models of disease, Proteomic analysis, Transmission electron microscopy, Lysosomes

## Abstract

CINAC-patients present renal proximal tubular cell lysosomal lesions which are also observed in patients experiencing calcineurin inhibitor (CNI) nephrotoxicity, suggesting that CINAC is a toxin-induced nephropathy. An alternative hypothesis advocates chronic dehydration as a major etiological factor for CINAC. Here, we evaluated histological and molecular changes in dehydrated versus toxin exposed rats. Wistar rats were divided in 3 groups. Group 1 (n = 6) had free access to drinking water (control group). Group 2 (n = 8) was water deprived for 10 h per 24 h, 5 days/week and placed in an incubator (37 °C) for 30 min/h during water deprivation. Group 3 (n = 8) underwent daily oral gavage with cyclosporine (40 mg/kg body weight). After 28 days, renal function, histopathology and proteomic signatures were analysed. Cyclosporine-treated rats developed focal regions of atrophic proximal tubules with associated tubulo-interstitial fibrosis. PASM staining revealed enlarged argyrophilic granules in affected proximal tubules, identified as lysosomes by immunofluorescent staining. Electron microscopy confirmed the enlarged and dysmorphic phenotype of the lysosomes. Overall, these kidney lesions resemble those that have been previously documented in farmers with CINAC. Dehydration resulted in none of the above histopathological features. Proteomic analysis revealed that dehydration and cyclosporine both induce injury pathways, yet of a clear distinct nature with a signature of toxicity only for the cyclosporine group. In conclusion, both cyclosporine and dehydration are injurious to the kidney. However, dehydration alone does not result in kidney histopathology as observed in CINAC patients, whereas cyclosporine administration does. The histopathological analogy between CINAC and calcineurin inhibitor nephrotoxicity in rats and humans supports the involvement of an as-yet-unidentified environmental toxin in CINAC etiology.

## Introduction

In the 1990s, clinicians in El Salvador and Sri Lanka noted an unusually high number of patients with chronic kidney disease of unknown etiology (CKDu) in their rural communities^1,2^. Clinically, these patients had minimal or low proteinuria and lacked association with hypertension, glomerulopathies, or other common causes of CKD^3,4^. Epidemiological investigations revealed that this disease was primarily affecting agricultural workers, mostly men, often living and working in hot tropical climates^3,5^. This disease’s terminology has diverse given names, mostly location based, including CKD of non-traditional causes (CKDnt), Uddanam nephropathy in India and Mesoamerican nephropathy (MeN) in Central America. Histopathological analysis of renal biopsies from Sri Lanka^6,7^, El Salvador^8^, Nicaragua^9^ and India^10^ showed varying degrees of proximal tubular atrophy, tubulointerstitial fibrosis, and cellular infiltration. Given these combined epidemiological and histopathological characteristics, this renal disease was also given the more inclusive name “chronic interstitial nephritis in agricultural communities” (CINAC). Since its discovery, CINAC cases have been reported in a growing list of countries, (sub)tropical as well as within the temperate climate within the European Community^11–13^.

Two major etiological hypotheses have been put forward; however scientific consensus has not been reached. One hypothesis states that CINAC is caused by repeated episodes of dehydration related to performing hard labor in increased ambient heat^14–16^. Alternatively, it has been proposed that CINAC might originate from repeated and/or chronic exposure to environmental nephrotoxins, such as agrochemicals, metals, metalloids, halogens, etc.^17–19^. Given the epidemiological constant of an agricultural environment, pesticides and fertilizers are an obvious suspect. However, the data also remain associative and mechanistic evidence is lacking^18,20^.

Recently, in addition to the classical unspecific features of tubular atrophy, fibrosis and infiltration, we discovered an intriguing histopathological feature in human renal CINAC biopsies consisting in enlarged (> 1.2 µm) dysmorphic lysosomes containing numerous dispersed round to oval aggregates, specifically in the proximal tubular cells (PTCs)^11^. These features contrasted with normal lysosomes which are generally smaller (0.1 to 1 µm) and spherical in shape with a rather homogeneous content. Interestingly, the aberrant lysosomes were absent or only scarcely present in “normal” human renal tissue as well as in several common nephropathies associated with CKD such as interstitial nephritis, glomerulonephritis, pyelonephritis, proteinuric nephropathies, minimal change disease, lupus and focal segmental glomerulosclerosis^11^. On the other hand, the lysosomal lesion was identified in several nephropathies mostly associated with exposure to nephrotoxins, such as lomustine, clomiphene, tenofovir and one particular type of light chain disease^11^. Strikingly, the CINAC-lysosomal phenotype was also observed in transplant patients exposed to immunosuppressive calcineurin-inhibitors (cyclosporine and tacrolimus), which feature a well-known inherent nephrotoxicity^11^. Although there is no evidence to date that calcineurin inhibition is directly involved in the development of CINAC disease, it is of note that renal calcineurin is abundantly expressed in the PTCs, the cell type most heavily affected in CINAC^21,22^. Also, calcineurin directly regulates Transcription Factor EB (TFEB), a genetic controller of the “coordinated lysosomal expression and regulation” (CLEAR) element which synchronises the expression of genes involved in many lysosomal processes^24–26^. Given the fact that the lysosomal lesion, up to now, is nearly exclusively shared with toxin-associated conditions and nephropathies, it is highly likely that development of CINAC requires the presence of a toxin^21–26^.

To contribute to our current etiological understanding, we here evaluated at multiple levels, i.e. functional, histological and molecular (proteomic), to what extent repetitive dehydration and cyclosporine exposure of healthy rats can injure the kidney and induce a CINAC-like (lysosomal) phenotype. Since the actual CINAC-inducing toxin(s) remain unidentified, cyclosporine was used as a relevant nephrotoxic control based on the previously documented histopathological similarity between CINAC and patients treated with calcineurin inhibitors.

## Results

### Animal physiology

**Figure 1:**
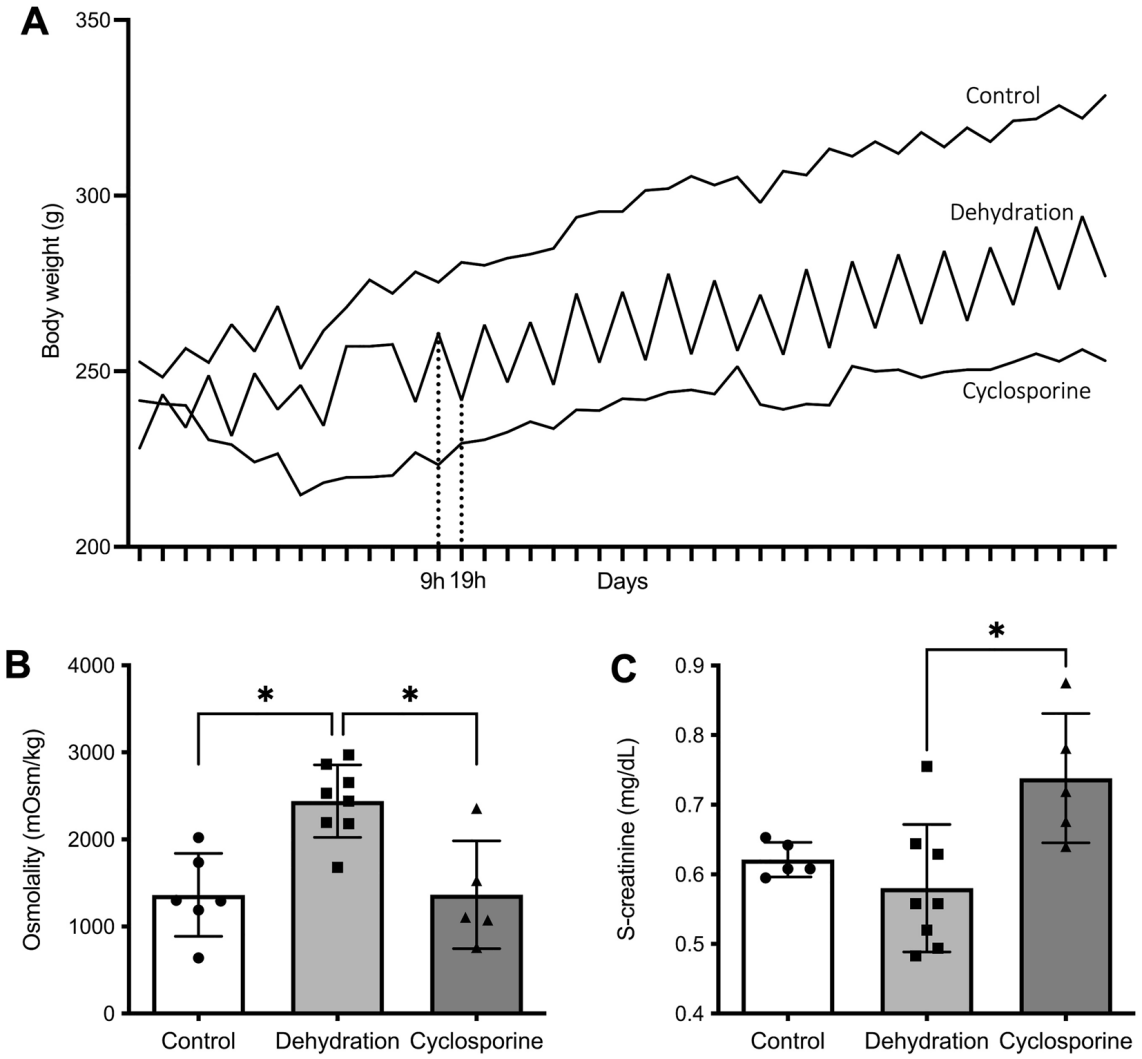
Clinical parameters. (**A**) Body weights were measured daily at the start (09h00) and end (19h00) of the water retention period. The daily recurrent drop reflects the dehydration vs. rehydration periods (marked with dotted line). (**B**) Osmolality measurements of urine of dehydrated animals, collected after 10 h of dehydration, compared to control and cyclosporine-exposed animals, collected after 24 h, at the end of the study period on week 4. (**C**) Serum creatinine concentrations in control, dehydrated, or cyclosporine-administrated rats measured at study endpoint at 4 weeks of treatment. Data represents individual serum creatinine values and means ± SEM. *P < 0.05.

The dehydration period resulted in a daily transient loss of ca. 7% of body weight. By the end of the study, the dehydrated group was at 85% of the body weight of the control group which had ad libitum access to water. In cyclosporine-exposed rats, body weight gain was even more reduced versus controls resulting in 23% lower body weight at the study endpoint (Fig. 1A). The number of animals in the cyclosporine group (n = 5) was reduced due to 3 rats dying towards the end of the study. Rats’ urine osmolality was found to be increased in dehydrated rats (2440 ± 138 mOsm/kg) as compared to control rats (1363 ± 177 mOsm/kg) (Fig. 1B). No significant increase was observed in urine-osmolality of the cyclosporine-treated animals (1365 ± 247 mOsm/kg). As assessed by serum creatinine levels, renal function decreased significantly after 4 weeks of cyclosporine administration (0.74 ± 0.04 mg/dl) whereas no effect was noted in the dehydrated animals (0.57 ± 0.02 mg/dl) as compared to controls (0.52 ± 0.02 mg/dl) (Fig. 1C). In the control group, one sample was excluded due to low quality. Urinary protein showed the absence of overt proteinuria in all three groups (Fig. S1).

### Cyclosporine exposure induces focal cortical atrophy and aberrant proximal tubular lysosomes

**Figure 2:**
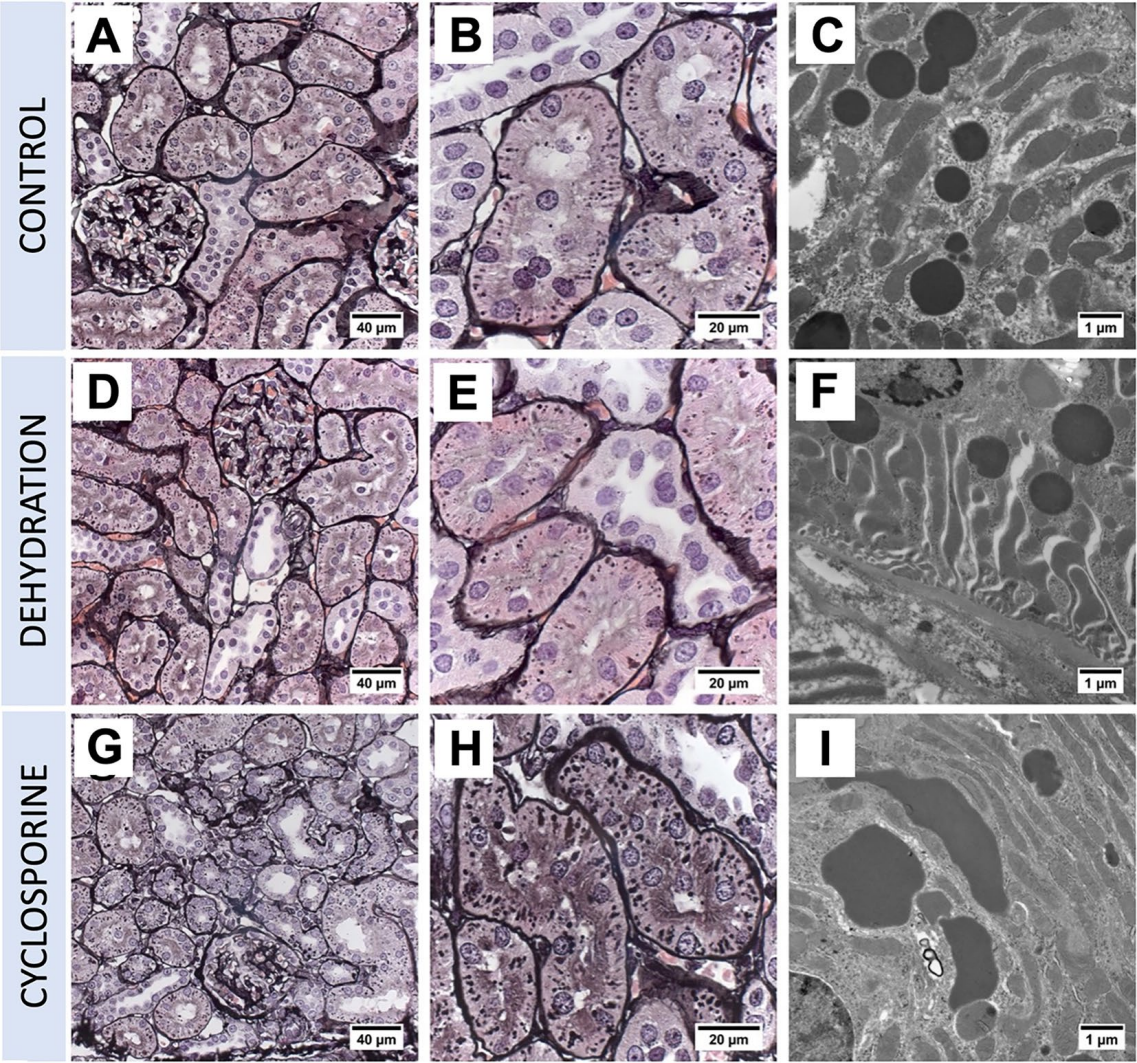
Renal histopathology in rats on PASM-silver stain and TEM of controls (**A**–**C**), dehydration (**D**–**F**) or cyclosporine exposure (**G**–**I**). PASM stain of cyclosporine administrated rat renal tissue showed focally dispersed atrophic regions of affected proximal tubular cells holding an increased number of enlarged and dysmorphic lysosomes (**G**,**H**). On TEM, these lysosomes are enlarged and dysmorphic (**I**). In control and dehydrated rats no PTC damage was observed (**A**,**B**,**D**,**E**) and lysosomes appear normal and spherical in shape on TEM (**C**,**F**).

**Figure 3:**
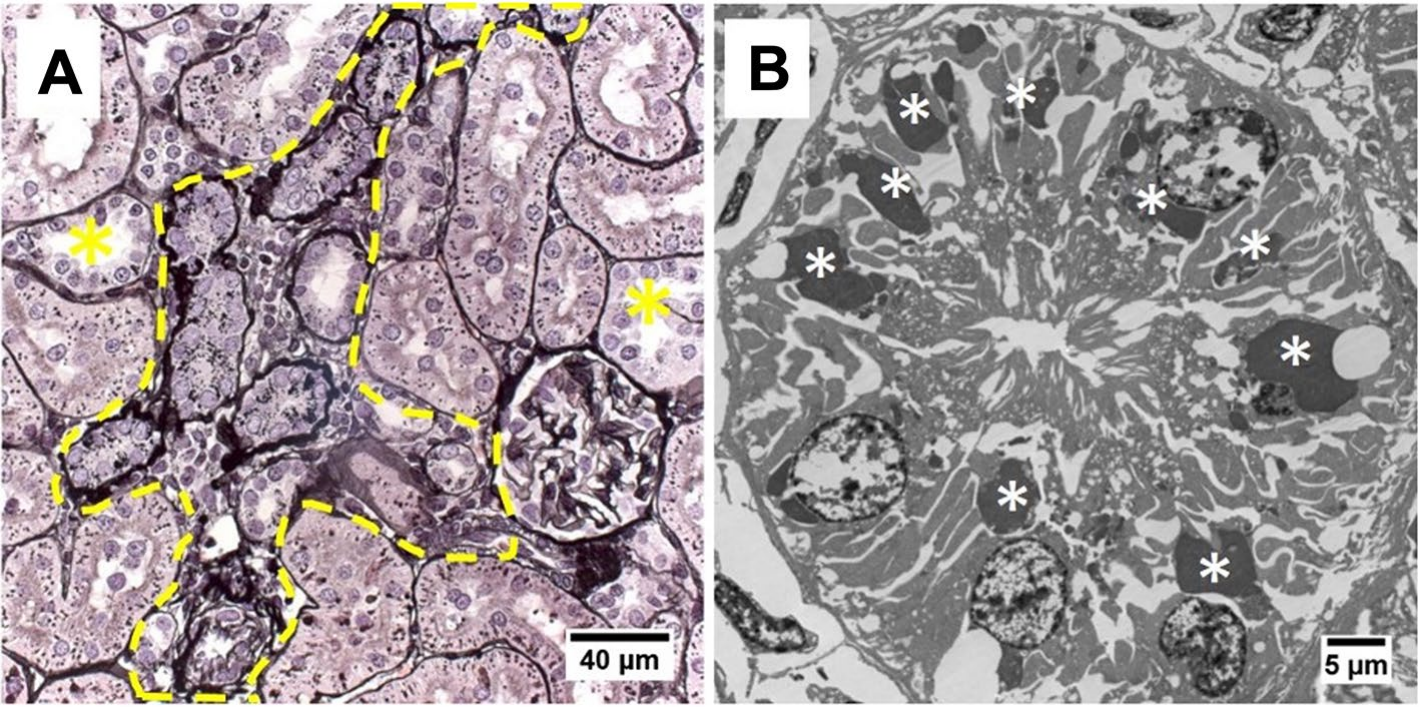
Renal histopathology of cyclosporine-exposed rats on PASM stain (**A**) and TEM of popoff paraffin-embedded tissue (**B**). PASM stain revealed affected atrophic PTC with an increased lysosomal number and thickened basement membrane with surrounded mild cellular infiltration (marked within yellow dotted line). Distal tubules (yellow asterisks) are unaffected. On TEM, prominent PTC dysmorphic lysosomes (white asterisks) appear as large as nuclei after CNI administration.

**Figure 4:**
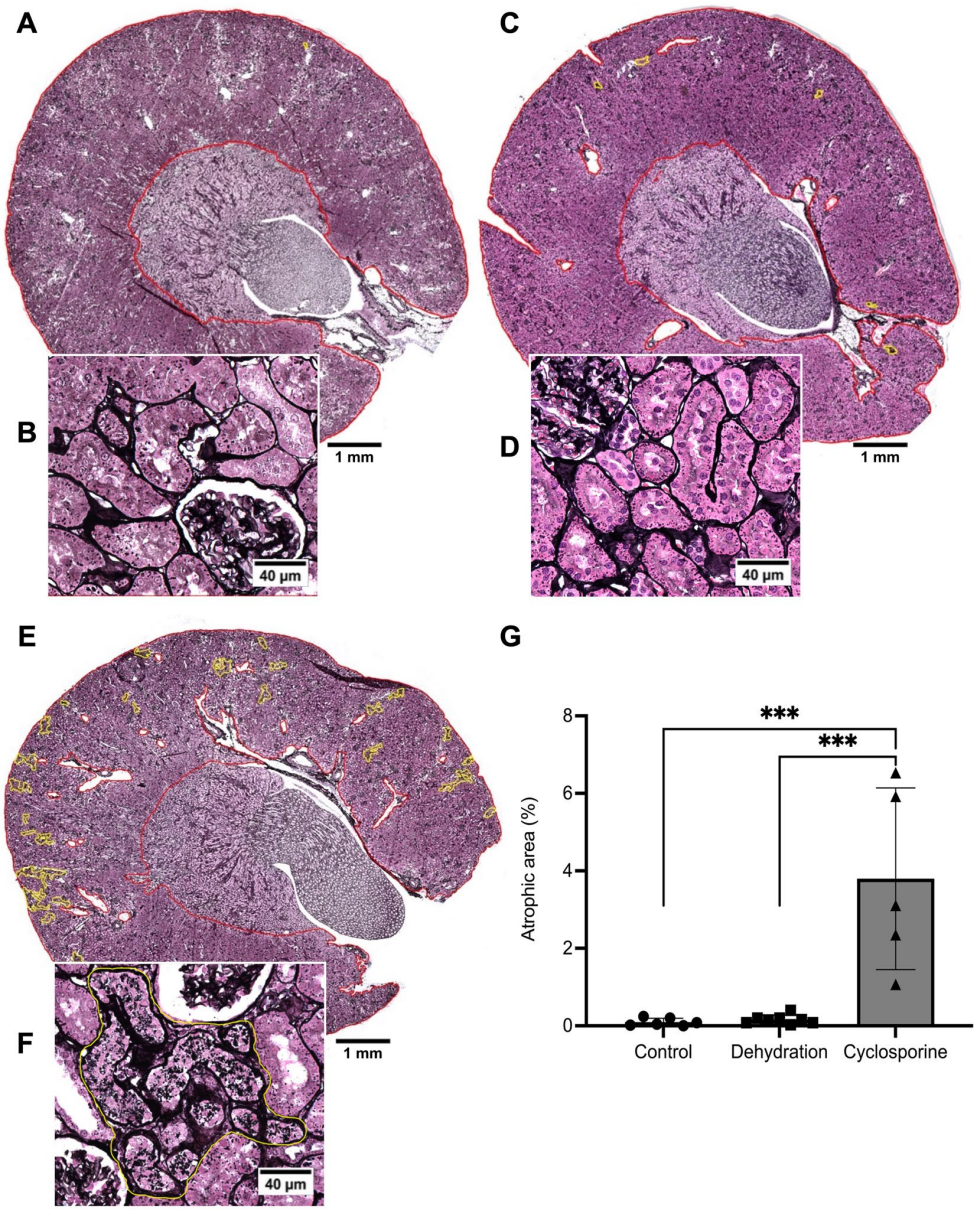
Evaluation of renal cortical atrophy. Whereas insignificant signs of atrophy were noted in control (**A**,**B**) and dehydrated (**C**,**D**) animals, atrophy clearly presented itself in the cyclosporin exposed group (**E**,**F**) in patches scattered throughout the cortex. The patches (marked by yellow lines) contain atrophic tubules with proximal epithelial cells filled with an increased number of enlarged argyrophilic granules in a context of mild interstitial fibrosis. (**G**) Area% of the combined atrophic patches over the delineated tissue (in red) consisting of cortex and the OSOM, but excluding medulla. Data presented as mean per animal ± SEM. ***P < 0.001.

**Figure 5:**
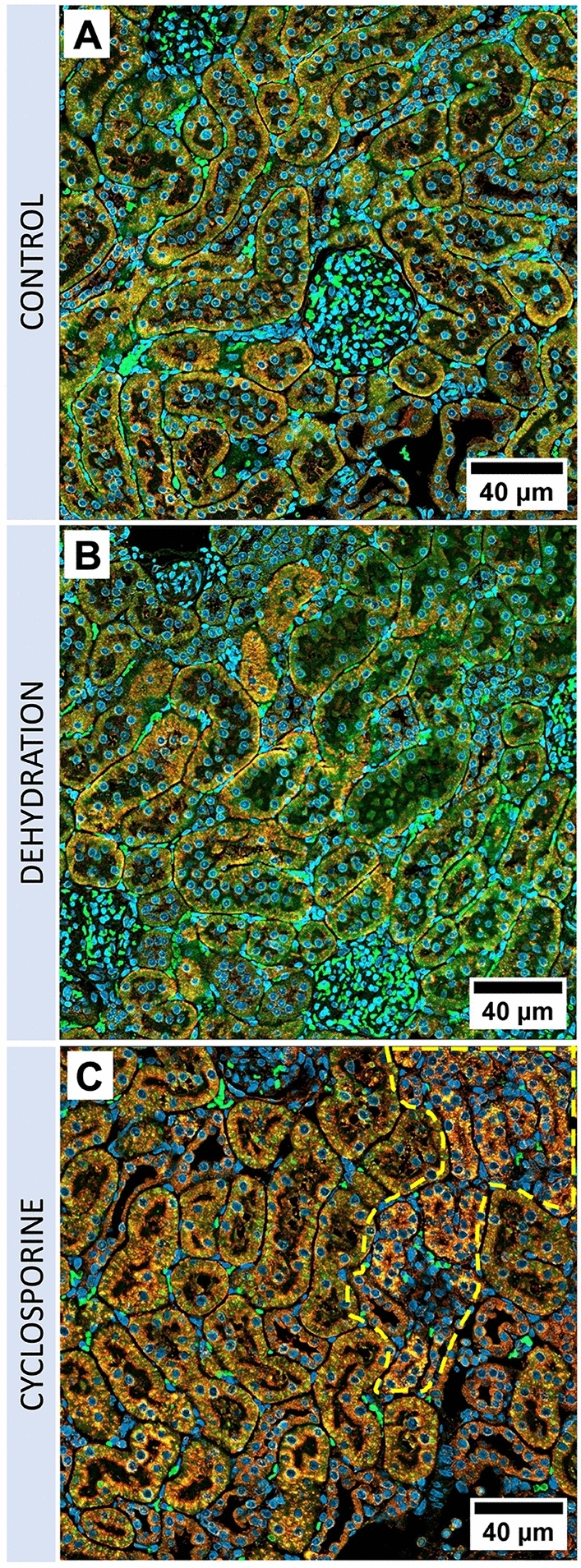
Immunofluorescence microscopy of control, dehydrated, and cyclosporine-administrated renal tissue. Fluorescence microscopy of red/yellow-stained lysosomal-associated membrane protein 1 (LAMP 1) demonstrating the increase of perinuclear lysosomal staining in proximal tubules of cyclosporine-administrated animals (**C**) as compared to control (**A**) and dehydrated (**B**) animals. Blue represents Hoechst-stained nuclei, green is emitted renal tissue autofluorescence. Affected atrophic PTCs with an increased lysosomal load are marked within the yellow dotted line.

**Figure 6:**
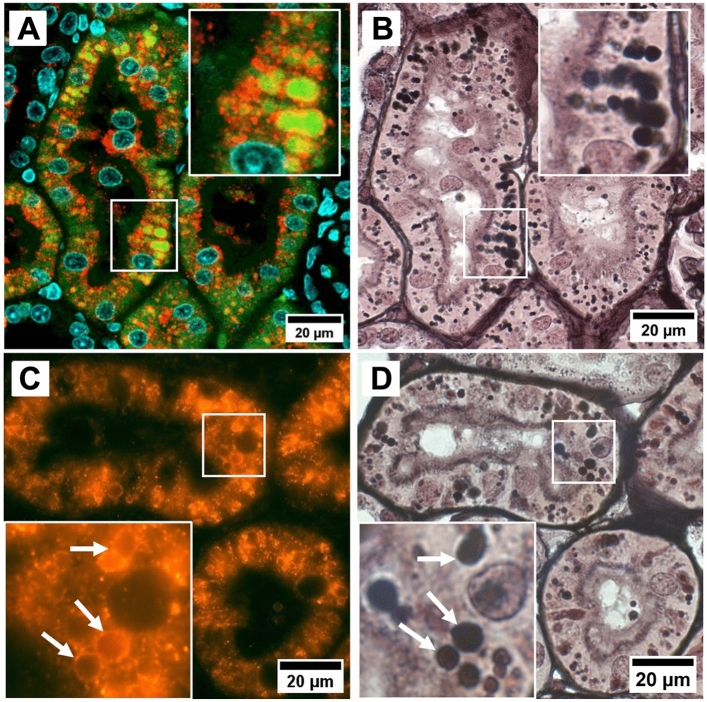
Enlarged intracellular granules are lysosomes. Immunofluorescent staining for red stained lysosomal-associated membrane protein 1 demonstrating enlarged green autofluorescent lysosomes in several proximal tubules in detail (**A**); Immunofluorescent red stained lysosomal-associated membrane protein 1 stain reveals large cytoplasmic granules are delineated by this lysosomal membrane marker in detail (white arrows) (**C**). PASM staining of same section (**B**,**D**) revealed that the same lysosomes are argyrophilic. Images are from a cyclosporine-administrated animal.

After 4 weeks of cyclosporine exposure renal histology showed cortical PTC injury consisting in focal regions of proximal tubular atrophy associated with brush border loss, thickening of basement membranes, with edema, mild interstitial cellular infiltration and fibrosis (Figs. 2G and 3A). Quantification of these atrophic zones revealed a combined cortical surface area% of 3.79 ± 2.34% in cyclosporine exposed animals which was significantly higher than in controls (0.02 + /–0.02%) (Fig. 4). No glomerular lesions were observed. In addition, Periodic Acid Silver Methenamine (PASM) staining demonstrates an increase in enlarged argyrophilic vesicles, previously identified as lysosomes^11^, in atrophic and intact PTCs (Fig. 2G,H). Fluorescent imaging for Lysosomal-associated Membrane Protein 1 (LAMP1) demonstrates an increased presence of enlarged lysosomes in the proximal tubules of cyclosporine-exposed animals as compared to controls (Fig. 5A,C). The nature and nephron location of these vesicles was reconfirmed by combining fluorescence imaging of LAMP1-stainined sections with subsequent PASM staining (Fig. 6). Transmission electron microscopy (TEM) revealed PTCs, with enlarged, occasionally dysmorphic lysosomes, comparable to those previously reported in CINAC and CNI-treated patients, however without the intralysosomal electron-dense round aggregates^11^ (Figs. 2I and 3B).

### Chronic dehydrated rats demonstrate normal proximal tubular histology

In dehydrated and control animals, no histological lesions were observed in proximal tubules nor glomeruli. Quantification of atrophic zones revealed no significant difference between dehydrated (0.16 ± 0.11%) and control animals (0.02 ± 0.02%) (Fig. 4). PTCs in both groups showed small scattered argyrophilic lysosomes on PASM staining (Fig. 2A,B,D,E) with a fairly round uniform appearance on TEM (Fig. 2C,F). No enlarged dysmorphic lysosomes, as those observed in cyclosporine exposed rats (Fig. 5C), were noted in PTCs of dehydrated and control animals (Fig. 5A,B).

### Dehydration and cyclosporine have highly distinct proteomic responses

**Figure 7:**
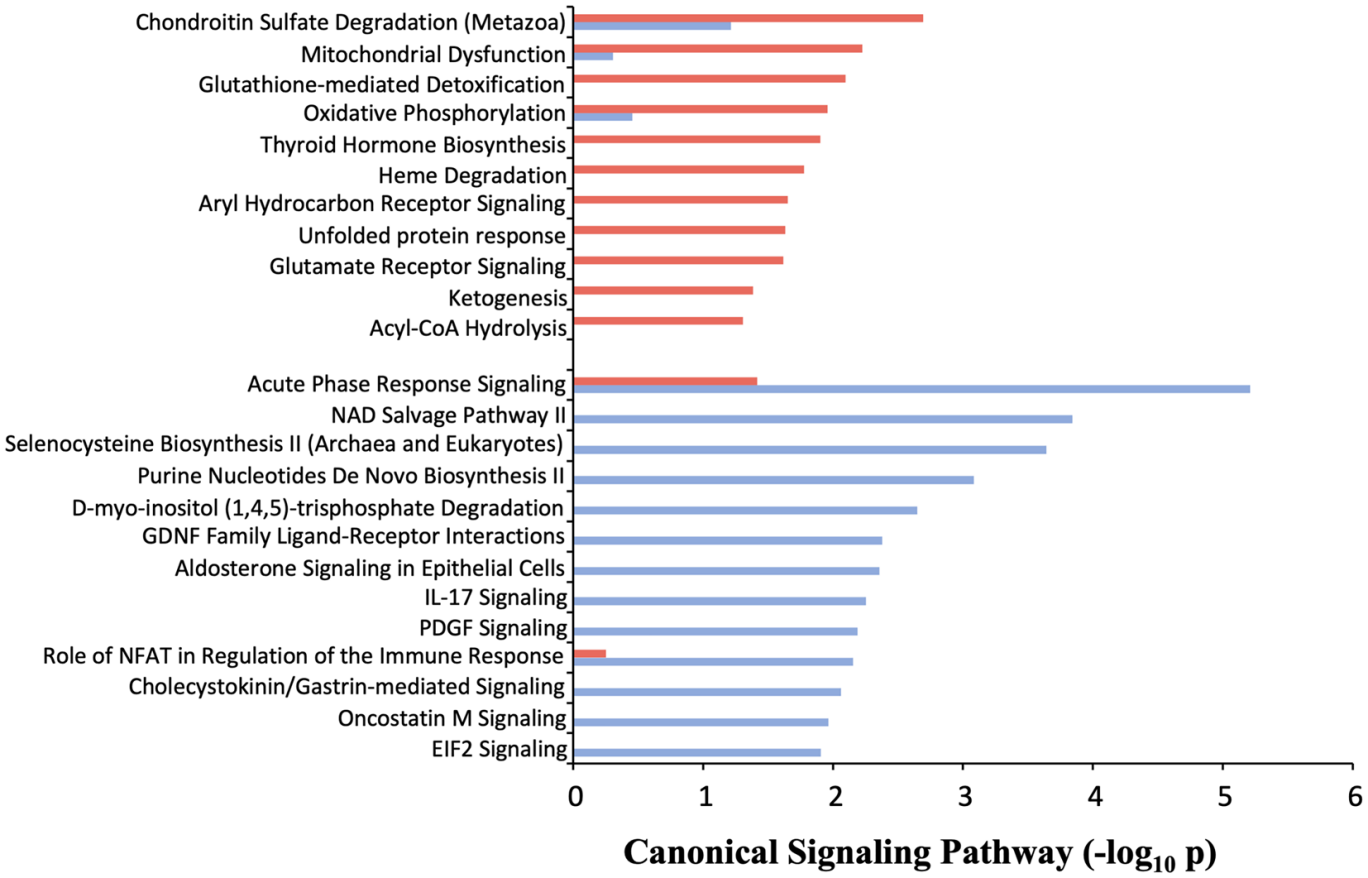
IPA canonical signalling pathway enrichment analysis of cyclosporine or dehydration DEP lists. IPA qualitative signalling pathway nature of both cohorts demonstrating cyclosporine exposure (red) associated with metabolic dysfunction pathways while dehydration (blue) is predominantly linked with inflammatory processes and vasculature-related receptor tyrosine kinase pathways.

In total 220 differentially expressed proteins (DEPs) were significantly altered: 108 in response to dehydration (37 upregulated and 71 downregulated compared to control rats, Table S1) whilst 112 in cyclosporine treated animals (65 upregulated and 47 downregulated compared to control rats, Table S2). Twenty-four proteins were common to both (12.2% of total), 17 of which were regulated with the same expression polarity compared to controls (7.6%) and 7 with opposing expression polarity between the two experimental paradigms (Fig. S2: Table S3). Canonical signaling enrichment analysis of the two DEP lists revealed a minimal enriched pathway commonality between the two datasets, i.e. 5.9% for Gene Ontology (GO)-terms and 2.4% for signaling pathways (Fig. S3, Tables S4 and S5). Pairwise data comparison in Ingenuity Pathway Analysis (IPA) showed that cyclosporine exposure was strongly associated with metabolic dysfunction (‘Mitochondrial Dysfunction’, ‘Oxidative Phosphorylation’, ‘Ketogenesis’, ‘Acyl co-A hydrolysis’) and stress-related pathways (‘Glutathione-mediated Detoxification’, ‘Unfolded protein response’), while dehydration was linked with inflammatory processes (‘Acute Phase Response Signaling’, ‘IL-17 Signaling’, ‘Oncostatin M Signaling’, ‘Role of NFAT in Regulation of the Immune Response’) and vasculature-related receptor tyrosine kinase pathways (‘GDNF Family Ligand-Receptor Interactions’, ‘PDGF Signaling’) (Fig. 7; Table S6).

### Dehydration and cyclosporine exposure responses are validated by their unbiased signature profiles

Dehydration and cyclosporine DEPs demonstrate highly distinct proteomic responses. These were validated by benchmark proteomic signatures based on terms relevant with the CINAC-mechanistic and histological phenotype. Signatures were cross matched with literature-derived available proteomics databases and terms were significantly linked to either dehydration or cyclosporine DEPs as compared to a randomly derived protein list. As such, the ‘dehydration’ overlapped more with DEPs from the dehydrated group (11 out of 603) while ‘cyclosporine’ benchmarked proteomic signature found the most overlap with DEPs from the cyclosporine exposure group (8 out of 305). Proteomic analysis also demonstrated that DEPs of the cyclosporine-treated group had significantly more overlap with the ‘lysosome’ (20 out of 112), ‘lysosomal storage disease’ (11 out of 654); ‘fibrosis’ (16 out of 1314); ‘senescence’ (10 out of 500) and ‘mitochondria’ (27 out of 2642) benchmarked proteomic signatures than dehydration did (Fig. S4, Table S7).

### Cyclosporine and dehydration induce distinct renal injury pathways

**Figure 8:**
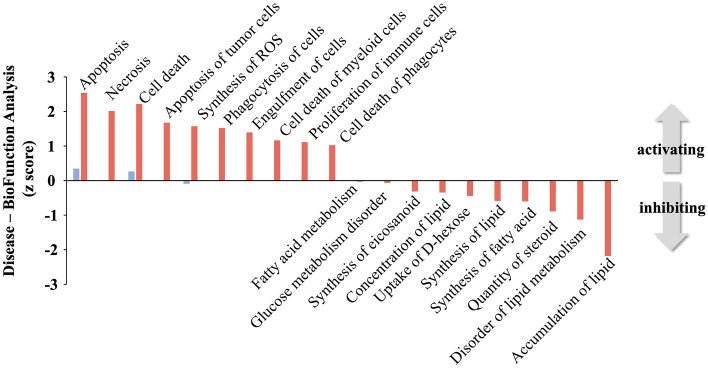
IPA-based disease & bio-function pathway enrichment analysis of cyclosporine or dehydration DEP lists. Cyclosporine administration in rats upregulates proteins (red) involved in processes linked to cellular pathology and oxidative stress, while downregulating proteins involved linked with fatty acid metabolism and glucometabolic dysfunction. Dehydration cohort proteins (blue) depict a minimal degree of classical disease/bio-function annotation, suggesting a less coherent molecular signalling response.

**Figure 9:**
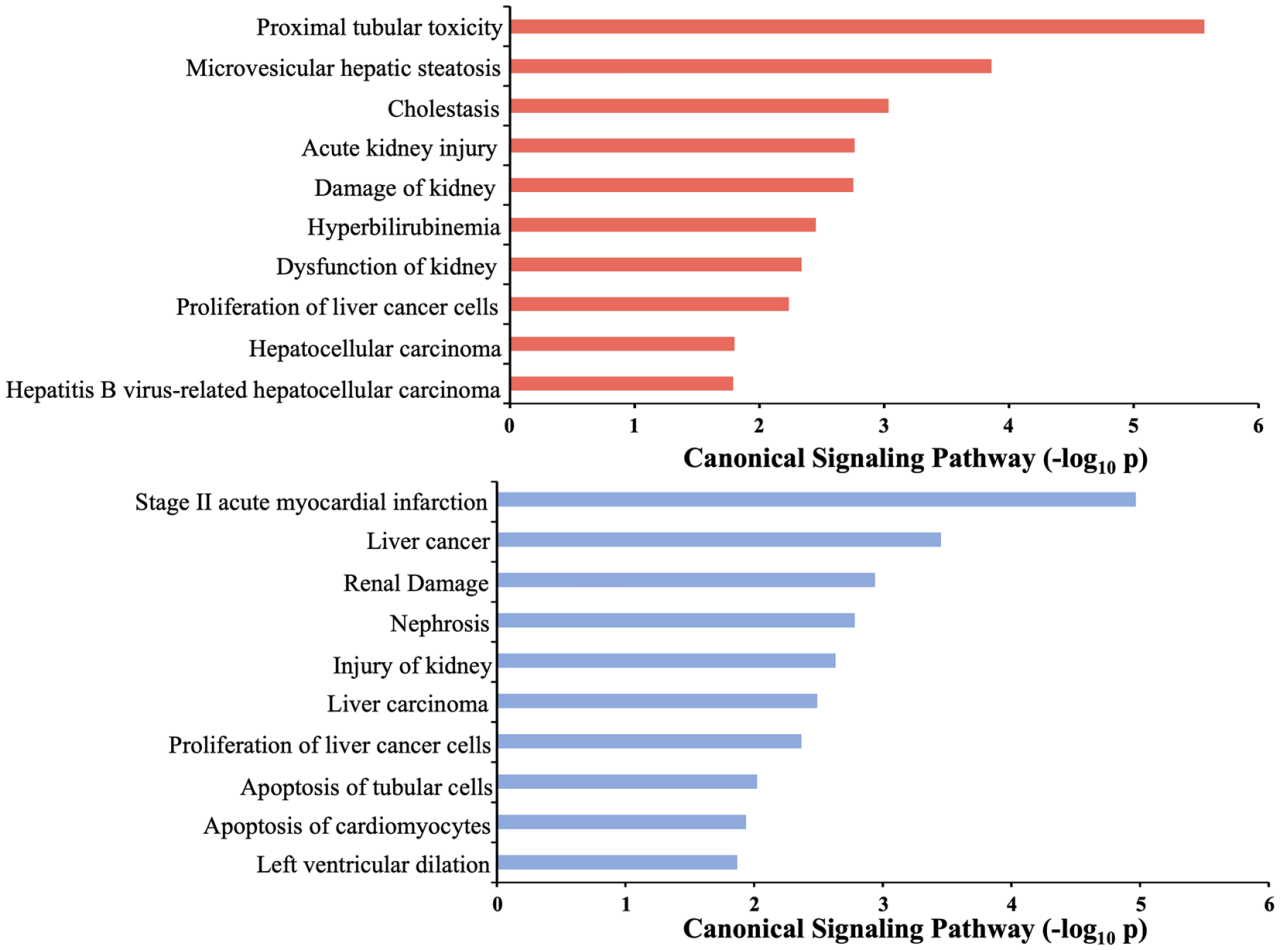
IPA-based toxicity pathway enrichment analysis of dehydration and cyclosporine. Both dehydration (blue) and cyclosporine (red) DEP cohorts demonstrate renal injury pathways through distinct proteomic cell signalling responses. Cyclosporine exposure is significantly associated to renal toxicity-induced injury pathways while dehydration is linked to renal damage/injury without a toxicity link.

Cyclosporine exposure positively populated (i.e. annotation based on upregulated proteins) multiple processes linked to cellular pathology (‘Apoptosis’, ‘Cell death’, ‘Necrosis’, ‘Synthesis of reactive oxygen species’), while negatively populated (i.e. annotation based on downregulated proteins) processes linked with fatty acid metabolism (‘Accumulation of Lipid’, ‘Disorder of Lipid Metabolism’, ‘Synthesis of Fatty Acid’, ‘Fatty acid metabolism’) (Fig. 8, Table S8). Both dehydration and cyclosporine cohorts demonstrated significant enrichment of renal damage-associated toxicity pathways (Fig. 8; Tables S9, S10). Cyclosporine exposure was associated with ‘Proximal tubular toxicity’, ‘Acute kidney injury’, ‘Damage of kidney’, ‘Dysfunction of kidney’ IPA-based toxicity terms, whereas dehydration was linked with ‘Renal Damage’, ‘Injury of Kidney’, ‘Apoptosis of tubular cells’ (Fig. 9).

### Identification of proteins underlying dehydration and cyclosporine responses

Textrous! analysis of dehydration data generated a text matrix with a dominant cluster of protein-word correlations linked to cytoskeletal reorganization as well as with a small cluster associated with oxidative stress (Table S11). Cyclosporine data analysis presented clusters linked to energy metabolism failure/oxidative stress and fatty acid metabolism (Table S12). Textrous! word cloud analysis corroborated the functional divergence between dehydration and cyclosporine effects (Fig. S5). Sub-cluster analysis demonstrated that for the dehydration matrix the following proteins were responsible for the bulk of the phenotype, i.e. Cttn, Mras, Trip4, Myo1, Myof, Scin, Unc45a, Podxl and Snx18. Sub-cluster analysis for cyclosporine identified main contributions for Acox1, Cox6b1 Cox7b, Blvra, Akr1c13, Mgam and Gfer.

## Discussion

CINAC is a kidney disease with globally rising prevalence. Since its first reports in the early 90-ies, the etiology of this pathology receives growing scientific interest in search for its etiology. In this study, we investigated two major etiological suspects “dehydration” and “nephrotoxin(s)” and demonstrated, for the first time on the proteomic level, that both factors incite injury pathways within 4 weeks of exposure, yet of a clear distinct nature. On the histopathological level, rats exposed to the nephrotoxic immunosuppressant cyclosporine demonstrate a histopathological phenotype resembling to that previously reported in CINAC patients, both by light and electron microscopy^7,11^ For dehydration this was not the case, suggesting that dehydration alone is less likely to induce CINAC.

The immunosuppressive CNI cyclosporine is a well-documented nephrotoxin that has been strongly associated with renal pathological alterations such as interstitial fibrosis, tubular atrophy and glomerulosclerosis^27–29^. A recent renal histopathological study by our group revealed that CINAC patients presented proximal tubular lesions (enlarged dysmorphic lysosomes) similar to those observed in (transplant) patients treated with immunosuppressant calcineurin inhibitors^11^. To the date of this publication, there is no literature available demonstrating the observed lysosomal and histopathological phenotype occurs in pesticide/environmental toxin exposed individuals. We here demonstrate that cyclosporine-treated rats present lesions comparable to human renal histological abnormalities consisting in areas of atrophic tubules with low-to-mild fibrosis/inflammation scattered throughout the cortex. Given this patchy nature and absence of glomerular lesions, we conclude that these features represent the initiating events of tubular CNI-toxicity. The non-significant trend of increased serum creatinine after 4 weeks cyclosporine exposure, as compared to controls, also corresponds with such an early onset of CNI pathology.

TEM examination of cyclosporine-exposed rat kidneys demonstrated a proximal tubular lysosomal phenotype characterized by a significant enlargement with varying degrees of dysmorphia, features that have also been reported in CINAC patients as well as in patients treated with CNI’s (Fig. S7)^11^. Enlarged lysosomes were also present in PTCs with intact brush borders, indicating that their appearance may occur prior to tubular atrophy. Whether their presence is mechanistically linked to eventual epithelial demise or whether it can be transient, remains to be determined. Furthermore, in contrast to human histopathology, the aberrant lysosomes in rats were without the dispersed intralysosomal round electron-dense aggregates. Nonetheless, both in experimental cyclosporine exposure and CINAC/CNI-treated patients the proximal tubular endolysosomal compartment is involved, suggesting important mechanistic similarities, while also advocating a toxicological etiology for CINAC.

In our rats with confirmed dehydration by transient decreases in body weight and increased urinary hyperosmolality, no histological abnormalities of CKD (atrophy, fibrosis, glomerulosclerosis), CINAC/CNI (enlarged dysmorphic proximal epithelial lysosomes) or acute kidney injury (massive epithelial cell loss) were noted. In a severe dehydration study of Macdonald et al., Wistar rats were deprived from water for 24, 48, and 50 h. As indicated by TEM, PTCs appeared normal and no lysosomal abnormalities were reported. Light microscopy (LM) analysis showed minor histological abnormalities comprising of thickened basement membranes and a slight dilatation of the glomeruli^30^. These, and our, histopathological findings are in contrast to a study in mice where 25 days (5 days/week) of recurrent dehydration led to mild PTC injury with some loss of the tubular brush border, and a mild degree of tubular fibrosis^31^. Yet, there are indications that mice are more susceptible to water deprivation than rats due to the body size difference^32,33^. In this context, it is worthwhile to note that the decrease in body weight after the dehydration period in our rats was ca. 7%, whereas in the above study in mice this was ca. 15%, indicating that the latter animals underwent more severe dehydration, which might explain the presence of the histopathological features in that model. However, to the best of our knowledge, letting alone whether it was specifically evaluated, the CINAC related lysosomal phenotype has not been spontaneously reported in any study where kidney effects of dehydration were investigated, neither in humans nor animals. Overall, the fact that severe repetitive dehydration (within the current 4-week time frame) does not lead to the histopathological spectrum which is shared between CNI-exposed rats on the one hand and CINAC/CNI-exposed patients on the other hand, further corroborates that CINAC has a toxicological origin. Additionally, epidemiological data from Sri Lanka, focusing on heat exposure, indicates that dehydration and heat stress alone do not exclusively contribute to the prevalence of CINAC disease, as evidenced by the fact that the population with the highest heat exposure exhibits a comparatively lower susceptibility to CINAC^34^. Also, the systematic review by Chapman et al. found no consistent evidence to support an association between CKD and heat-stress/dehydration while statistically significant associations were found for the use of pesticides^18^.

Proteomics made clear that both dehydration and cyclosporine cohorts demonstrated significant enrichment of renal damage-associated toxicity pathways. The IPA-derived toxicity terms of DEPs from the dehydration set that populate those pathways, however, are not specifically functionally linked to, nor considered a toxic phenotype. Cyclosporine derived IPA toxicity terms are effectively linked with a toxicological insult. This finding thus suggests that both insults are likely to incur renal damage but presumably through qualitative distinct pathways. Proteomic analysis thus confirms true cellular toxicity of cyclosporine, whereas the dehydration lacks any of such specific markers (Fig. S6). While the use of curated GO or signaling pathway databases can generate a functional appreciation of complex data sets recent advances in mass level natural language processing of biomedical text corpi has revealed that techniques such as latent semantic analysis (LSA) can generate more nuanced and more specific data-dependent appreciations of high-dimensionality data^35–37^. Using this approach with the natural language processing platform Textrous!, we found that dehydration associated text matrices revealed a clustering of proteins with terms predominantly associated with cytoskeletal reorganization as well as a distinct small cluster associated with oxidative stress. In strong contrast to this the cyclosporine DEP list generated a text matrix dominated by protein-word correlations linked to energy metabolism failure/oxidative stress and fatty acid metabolism, the main energy source in PTCs^38^. Using Textrous! extended noun-phrase outputs we were able to create high-dimensionality word clouds that again demonstrated the functional divergence between dehydration and cyclosporine effects (Fig. S5). Using a similar filtering approach here it was evident that for the dehydration text matrix the following proteins were responsible for the bulk of the rendered text phenotype, i.e. Cttn, Mras, Trip4, Myo1, Myof, Scin, Unc45a, Podxl and Snx18. Taken together these proteins demonstrate a strong link with renal cytoskeletal remodeling (Cttn, Myo1e, Scin, Un45a, Snx18) or renal injury and carcinogenesis (Trip4, Myof, Podxl)^39–46^. These data suggest that in response to dehydration there are multiple alterations in the subtle architecture of kidney that are likely affected to adjust to the reduced blood volume of the experimental animals. In contrast to this skeletal remodeling phenotype, the response to cyclosporine was again associated with a strong metabolic dysfunction (Acox1, Cox6b1 Cox7b, Blvra, Akr1c13), renal injury (Acox1, Mgam) and autophagy regulation (Gfer)^47–54^. Using this nuanced interpretation of complex data thus demonstrates that cyclosporine induces a strong metabolic/pro-aging phenotype in renal tissue that is highly distinct to that seen with dehydration. Considering the proteomic analysis of rat cortical renal tissue, it is interesting to note that regucalcin (Rgn) has been identified as an important renal calcium regulatory factor linked to the control of anti-apoptotic pathways^55,56^. We found that Rgn was downregulated in cyclosporine-treated rats compared to dehydrated animals and would therefore indicate a potential vital role in mediating kidney damage or failure in these animals. This finding is also supported by further analogous research into nephrotoxicants/immunosuppressives where Rgn downregulation is unequivocally associated with toxin-induced proximal tubular injury^57,58^. The benchmarked DEPs present a cyclosporine-specific, unique proteomic signature which correlates to a functional divergence, much greater than in the dehydrated group. As such, it demonstrates that in cyclosporine-treated animals the processes of altered lysosomal function and cellular senescence are occurring in the cortical area of the kidney. Importantly, Kolli et al. recently were the first to publish on the urinary proteome of CKDu/CINAC patients, reporting findings similar to ours, e.g. roles of mitochondrial, lysosomal, and protein reabsorption processes in disease onset and progression of CINAC^59^.

This study has limitations. No animal group was included that was both dehydrated and exposed to cyclosporine. Hence, we cannot conclude on dehydration either or not being an aggravating factor in the development of CINAC. Furthermore, this study was conducted over a 4-week period, which is rather short in translational comparison to years of exposure in humans. Yet, even in this study, differences were clear.

In conclusion, these data suggest a clear difference between tubular damage due to dehydration and damage due to a toxic insult. The fact that cortical PTCs and lysosomal lesions induced by cyclosporine in rats closely resembles CINAC pathology, whereas dehydration alone does not, supports a toxicological etiology for CINAC and makes it unlikely that dehydration by itself is the main causal driver. However, this does not exclude the possibility for dehydration to act as a sensitizing (e.g. increasing the susceptibility of PTCs to toxic insults), or aggravating factor in conditions of chronic toxin exposure^18^.

## Materials and methods

### Animals

This study was conducted in eight-week-old male albino rats of the Wistar strain (Charles River, France). Rats acclimatized for 7 days and were randomly divided in three groups. Group 1 (n = 6) received water ad libitum (control group). Group 2 (n = 8) was deprived of water for 10 h per 24 h for 5 days a week, during 4 weeks. During water deprivation, rats were placed in an incubator at 37 °C for 30 min each hour. Group 3 (n = 8) was administered 40 mg/kg body weight of cyclosporine (Neoral-Sandimmun, Novartis) by daily oral gavage for a total study duration of 4 weeks. Cyclosporine dose initiated at 50 mg/kg body weight but was reduced to 40 mg/kg after 10 days into the study due to mortality of 3 animals. Rats’ bodyweight was recorded daily. Rats were placed in metabolic cages for 24 h on day 3, 17, and 28 after the start of dehydration and cyclosporine exposure. The osmolality of urine samples, measured by freezing point depression using OsmoPro (I&L Biosystems) was determined (10-h urine collection immediately after the dehydration period, 24-h urine collection of control and cyclosporine-treated animals) and results are expressed as mOsm/kg. Blood was taken from tail vein for serum creatinine measurement using the colorimetric Jaffé method, the results are expressed as mg/dl. The urinary protein concentration was measured by standard Bradford protein assay, results are expressed as mg/ml. During their 24 h housing in metabolic cages, no animals were exposed to increased environmental temperature. After 28 days of dehydration or cyclosporine exposure, all rats were euthanised by exsanguination through the retro-orbital plexus under deep anesthesia using ketamine/xylazine, and kidneys were collected for histological- and proteomic analyses. Dehydrated rats were euthanised immediately after the water deprivation period. Experimental procedures were conducted according to the National Institutes of Health Guide for the Care and Use of Laboratory Animals and approved by the Ethical Committee of the University of Antwerp (Agreement number ECD2018-92) and were in accordance with ARRIVE guidelines^60,61^.

### Light microscopic histological evaluation

Collected kidney specimens were fixed in 4% paraformaldehyde, embedded in paraffin, and cut into 2 µm and 4 µm-thick sections. Using LM, histopathologic examination of renal damage was performed on PASM-stained renal tissue sections. For immunofluorescent staining LAMP1 (ab24170, Abcam, 1:200 dilution) was used.

Using QuPath (version 0.3.1) imaging processing software, on whole renal section images, proximal tubular atrophy and tubulointerstitial fibrosis, focally dispersed in areas across the renal cortex areas, were semi quantified on PASM stained sections. Areas of the cortical and outer medulla region of the renal section were delineated and combined atrophic zones were calculated with results expressed as area%.

### Electron microscopic histological evaluation

Samples were first fixated in glutaraldehyde followed by subsequent post-fixation in OsO4 after which they were embedded in epoxy resin. Reprocessing of formalin fixated paraffin embedded samples involved deparaffinization, rehydration and embedding in epoxy resin. Ultrathin (50 nm) sections were collected on membrane-covered copper grids, counterstained with lead (with and without uranyl acetate) and carbon coated. Electron microscopes used included Zeiss, Fei and JEOL (100 CX) platforms.

### Quantitative tissue proteomics

Mass spectrometry and quantitative proteomics (using iTRAQ labeling) were performed on dissected cortical kidney tissue samples of control, dehydrated and cyclosporine exposed animals as described previously^62^. Kidney cortical tissue samples were ground completely in a protein extraction buffer (8 M urea, 2 M thiourea, 0.1% SDS in 50 mM triethylammonium bicarbonate solution). The concentrations of the extracted proteins were assessed using the reducing agent and detergent compatible (RCDC) protein assay kit (Bio-Rad, Hercules, CA, USA). Before trypsin digestion, equal amounts of protein from each sample were reduced and alkylated by tris-2-carboxyethyl phosphine and 5-methyl-methanoethiosulphate, respectively. The resulting peptides from each sample were labeled using iTRAQ reagents (AB-Sciex, Framingham, MA, USA) according to the manufacturer's instructions. To improve LC–MS/MS proteome coverage, samples were subjected to a 2D-LC fractionation system (Dionex ULTIMATE 3000, ThermoScientific, Waltham, MA, USA). The mixed peptides were first fractionated on a strong cation exchange chromatography polysulfoethyl aspartamide column (1 × 150 mm, (Dionex)) and secondly separated on a nano-LC C18 column (200 Å, 2 μm, 75 μm × 25 cm (Dionex)). The nano-LC was coupled online to a Q-Exactive™-Plus Orbitrap (ThermoScientific) mass spectrometer. The nano-LC eluents were infused to the Orbitrap mass spectrometer with a capillary at 1.7 kV on a nanoelectrospray ionization (nano-ESI) source at a flow rate of 300 nL/min. Data-dependent acquisition in positive ion mode was performed for a selected mass range of 350–1800 m/z at MS1 level (140,000 resolution) and MS2 level (17,500 resolution). The raw data were analyzed by Proteome Discoverer 2.0 software (ThermoScientific) using Sequest HT as search engine against the rattus norvegicus UniProt/SwissProt database with a threshold of confidence above 99% (false discovery rate less than 1%). Next, the list of identified proteins containing iTRAQ ratios of expression levels over control samples was generated.

### Bioinformatic analysis of proteomic data

#### Generation of differentially expressed protein lists

To identify the significantly altered proteins (i.e. proteins differentially expressed due to dehydration or cyclosporine exposure versus controls), raw iTRAQ ratios (dehydration or cyclosporine over control) were first log_2_ transformed. Next, DEP lists were created by identifying only proteins that possessed log_2_-transformed iTRAQ ratios two standard deviations (p < 0.05) from the calculated mean background expression variation level^63^.

#### Gene ontology and pathway analysis

We then investigated the proteomic divergence between dehydration and cyclosporine insults through IPA-based signaling pathway analyses, which can analyze the gene expression patterns using a build-in scientific literature-based database (www.ingenuity.com) and GO-term enrichment, the world’s largest source of information on gene function. In IPA, qualitative signaling pathways were identified by using the pairwise data comparison function. Significant signaling pathway population was only investigated for pathways significantly-populated with n > 2 proteins per IPA Canonical signaling pathway at an enrichment probability of p < 0.05. Similar criteria were also applied when using IPA for Disease/BioFunction and Toxicity database analyses. GO-term enrichment was performed with similar criteria using the Enrichr analytical suite (https://amp.pharm.mssm.edu/Enrichr/).

#### Generation of unbiased gene expression signature profiles from literature

Unbiased protein signature lists were generated by linking the terms ‘dehydration’, ‘cyclosporine’, ‘lysosome’, ‘lysosomal storage disease’ ‘senescence’, ‘mitochondria’ and ‘fibrosis’ through current database available systemic proteomic literature. Hence functional protein lists were generated with GLAD4U (http://glad4u.zhang-lab.org/index.php), GeneShot (https://maayanlab.cloud/geneshot/) and PubPular (https://heart.shinyapps.io/PubPular/) in a similar manner to our previous theoretical dataset constructs^64–66^. These terms were chosen based on the observed (immuno)histological phenotype, also previously described in Vervaet et al.^11^ in CINAC and CNI-treated patients with extensive proximal tubular atrophy, minimal proliferation or apparent apoptosis^11^. The multiple database-derived proteomic signature list for ‘dehydration’ consists out of 603 proteins ‘cyclosporine’ yielded 305; ‘lysosome’ 1668; ‘lysosomal storage disease’ had 645; ‘fibrosis’ had 1314; ‘mitochondria’ yielded 2642 proteins; and ‘senescence’ yielded a topped off 500 proteins. Dehydration and cyclosporine DEP lists from our rat study were cross referenced with these above-mentioned literature-derived signature lists and compared to an arbitrary software generated random protein list. This allows to evaluate to what extent dehydration and cyclosporine exposed rats present an expression pattern that overlaps with the expression signatures characteristic for the above selected terms, hence putative mechanistic involvement.

#### Identification of crucial proteins in response to cyclosporine or dehydration

Latent semantic analysis (LSA) of the dehydration and cyclosporine DEP lists was performed using *Textrous!* (https://textrous.irp.nia.nih.gov/), as previously described^67^. LSA is a computational investigation technique of the natural language capable of elucidating connections between interrogator concepts (e.g. proteins) and biomedical terms. *Textrous!* was applied to generate text matrices of protein-word correlations as well as word clouds by applying its ‘noun-phrase chunking’ function, in which biomedical phrases that are statistically associated (p < 0.05) with the input Gene Symbol lists are used. To uncover crucial proteins that underpin the complex physiological processes of exposure to cyclosporine or dehydration sub-cluster analysis of these matrices was performed^68^.

### Statistical analysis

Data are given as mean ± SEM. All statistical analysis was performed using Kruskal–Wallis and Mann–Whitney *U* test in GraphPad Prism, version 6.0c. P values < 0.05 were considered statistically significant.

### Supplementary Information

Supplementary Figures.

Supplementary Tables.

## Data Availability

The mass spectrometry proteomics data have been deposited to the ProteomeXchange Consortium via the PRIDE partner repository with the dataset identifier PXD038062. ProteomeXchange title: Rat Kidney MS analysis with dehydration and cyclosporine treatment. ProteomeXchange accession: PXD038062. Project Webpage: http://www.ebi.ac.uk/pride/archive/projects/PXD038062. FTP Download: ftp://ftp.pride.ebi.ac.uk/pride/data/archive/2022/11/PXD038062.
