# Supplementary material for: Chronic dehydration induces injury pathways in rats, but does not mimic histopathology of chronic interstitial nephritis in agricultural communities
Source: Sci Rep. 2023 Oct 23;13:18119. doi: 10.1038/s41598-023-43567-z (PMC10593944; doi:10.1038/s41598-023-43567-z)
Supplement: Supplementary file 1 — Supplementary Figures. [file 41598_2023_43567_MOESM1_ESM.pdf]

## Supplementary Figures

## Table of Contents

Figure S1: Urinary protein concentration of control, dehydrated and cyclosporine-administrated animals

Figure S2: iTRAQ quantitative proteomics identified DEPs in the dehydration and cyclosporine-treated cohorts.

Figure S3: Proteomic divergence between cyclosporine and dehydration insults through GO-term enrichment (a) and IPA-based (b) signaling pathway analyses.

Figure S4: Benchmarked protein signatures.

Figure S5: *Textrous!*-based high-dimensionality wordclouds of noun-phrase outputs of the functional divergence between dehydration (a) and cyclosporine (b) effects.

Figure S6: DEPs invalidating toxicity pathways in the dehydration and cyclosporine cohort.

Figure S7: Renal biopsies of transplant patients after cyclosporine- (A-C) and tacrolimus-treatment (D-F).

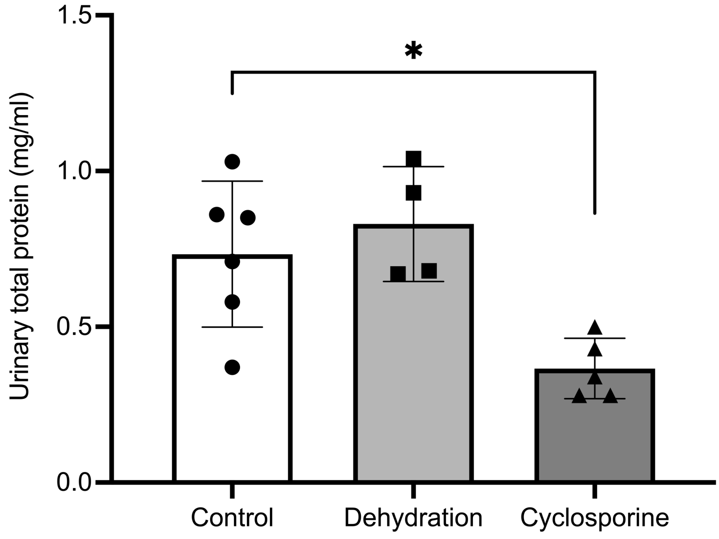


**Figure S1. Urinary protein concentration of control, dehydrated, and cyclosporine-administrated animals.** Bradford assay performed on rat 24h-urine collection to determine protein concentration. Data represents individual protein concentrations and mean ± SEM. * P < 0.05.

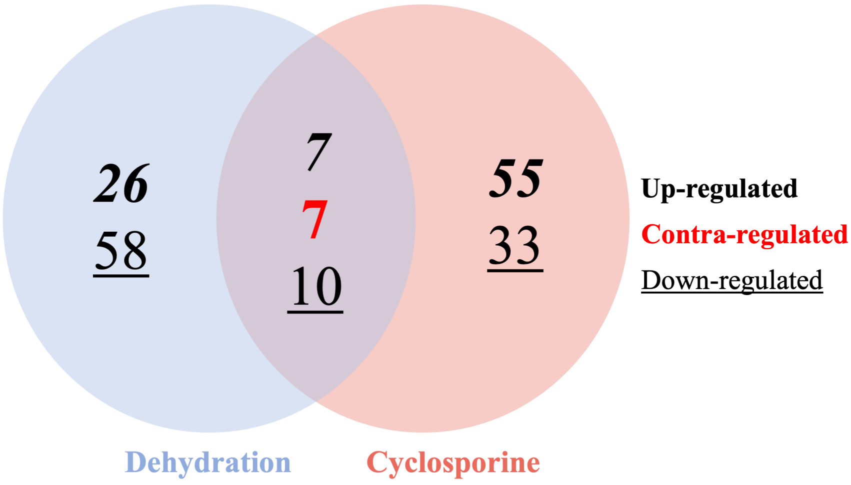


**Figure S2. iTRAQ quantitative proteomics identified DEPs in the dehydration and cyclosporine-treated cohorts.** A total of 220 DEPs are differentially expressed: 108 (37 up- and 71 downregulated) in response to dehydration and 112 (65 up- and 47 downregulated) in cyclosporine exposed animals. Twenty-four DEPs are common to both cohorts, of which 17 are regulated with same (7 co-upregulated; 10 co-downregulated), and 7 with opposing expression polarity.

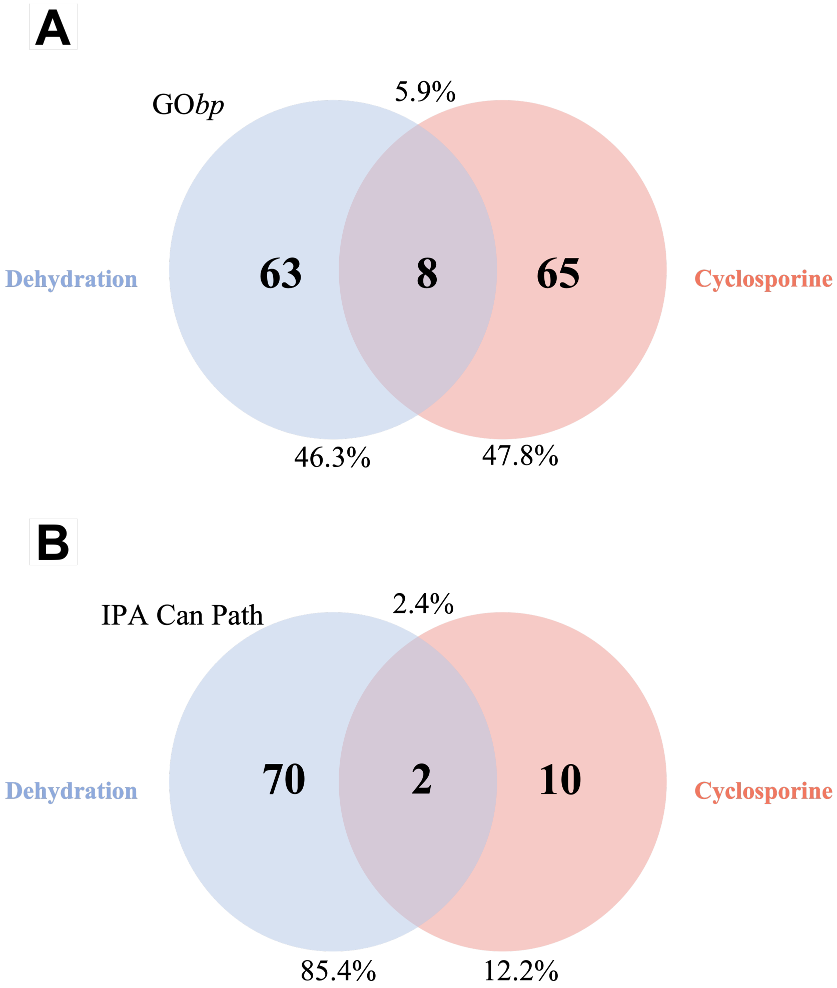


**Figure S3. Proteomic divergence between cyclosporine and dehydration insults through GO-term enrichment (A) and IPA-based (B) signaling pathway analyses**. While the two insults employed in this study (Cyclosporine or Dehydration) induced renal damage and stress their mechanistic activities were strongly divergent. Hence the Gene Ontology (biological process: GObp) and Canonical Pathway (Can Path) enrichment analysis of the Cyclosporine or Dehydration proteomic DEP lists demonstrated minimal levels of overlap. Using the input protein DEP lists we found, using GO (biological process domain) term annotation, there was only a 5.9% commonality of significantly-populated ($n>2$ proteins per GO term at $p<0.05$) GO terms, compared to the 7.6% protein identity level. This percentage commonality between dehydration and cyclosporine response datasets was even smaller when significantly-populated ($n>2$ proteins per IPA Canonical Signaling Pathway at $p<0.05$) signaling pathway analysis was employed, *i.e.* only a 2.4% pathway commonality was seen between the two proteome datasets. Therefore it is clear at the level of significantly altered proteins, clustered GO terms as well as canonical signaling pathways that dehydration and cyclosporine systemic effects in the kidney are highly distinct.

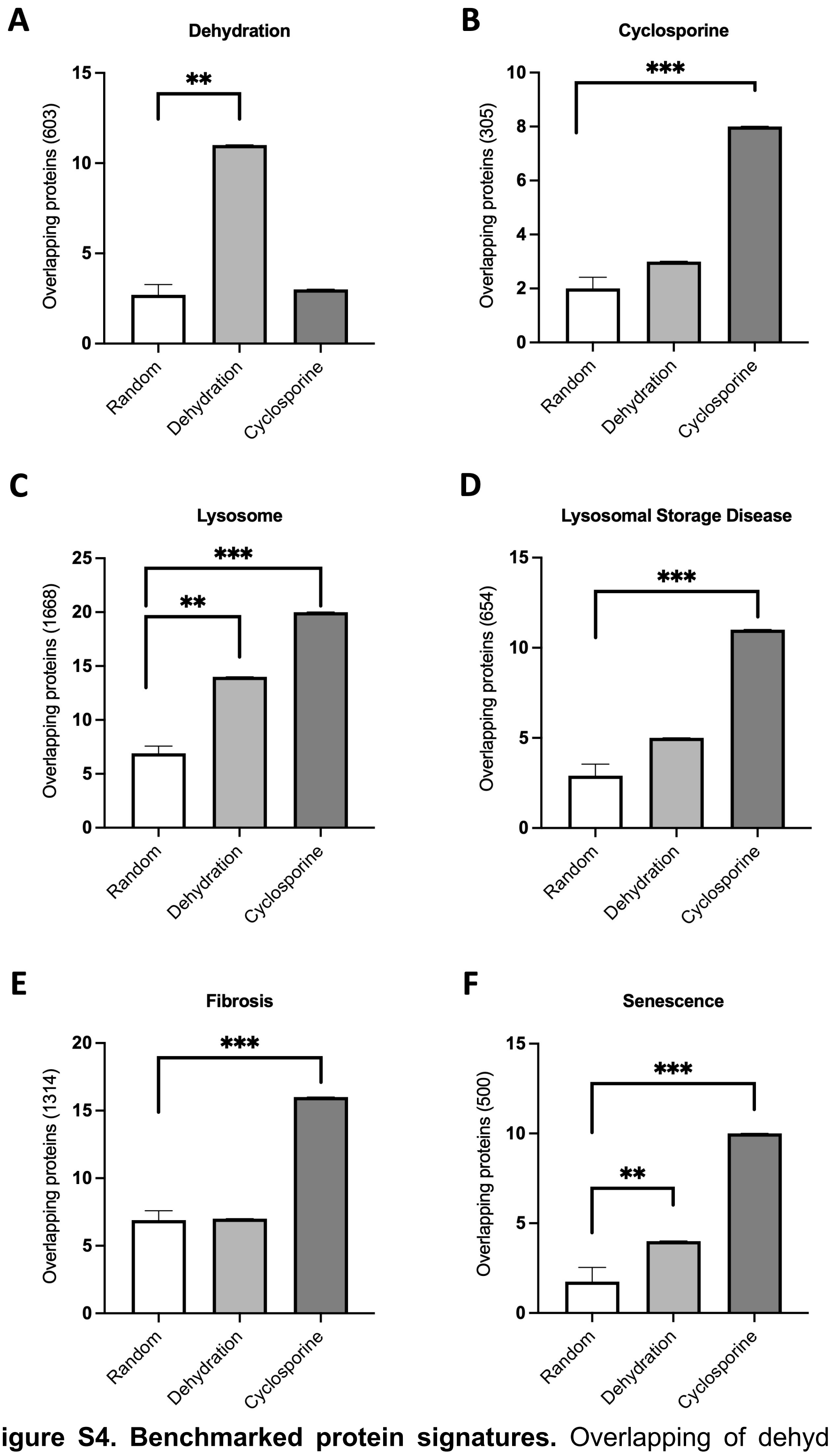


**Figure S4. Benchmarked protein signatures.** Overlapping of dehydration- and cyclosporine exposure-induced DEPs with literature-derived protein lists associated (extracted using Natural Language Processing analysis: GLAD4U - http://glad4u.zhang-lab.org/; GeneShot - https://maayanlab.cloud/geneshot/; PubPular - https://heart.shinyapps.io/PubPular/) with the terms 'dehydration'(n=603); 'cyclosporine'(n=305); 'lysosome'(n=1668); 'lysosomal storage disease'(n=654); 'fibrosis'(n=1314) and 'senescence'(n=500) compared to a randomly appeared protein list (n=112). P < 0.01(**); P < 0.001(***).

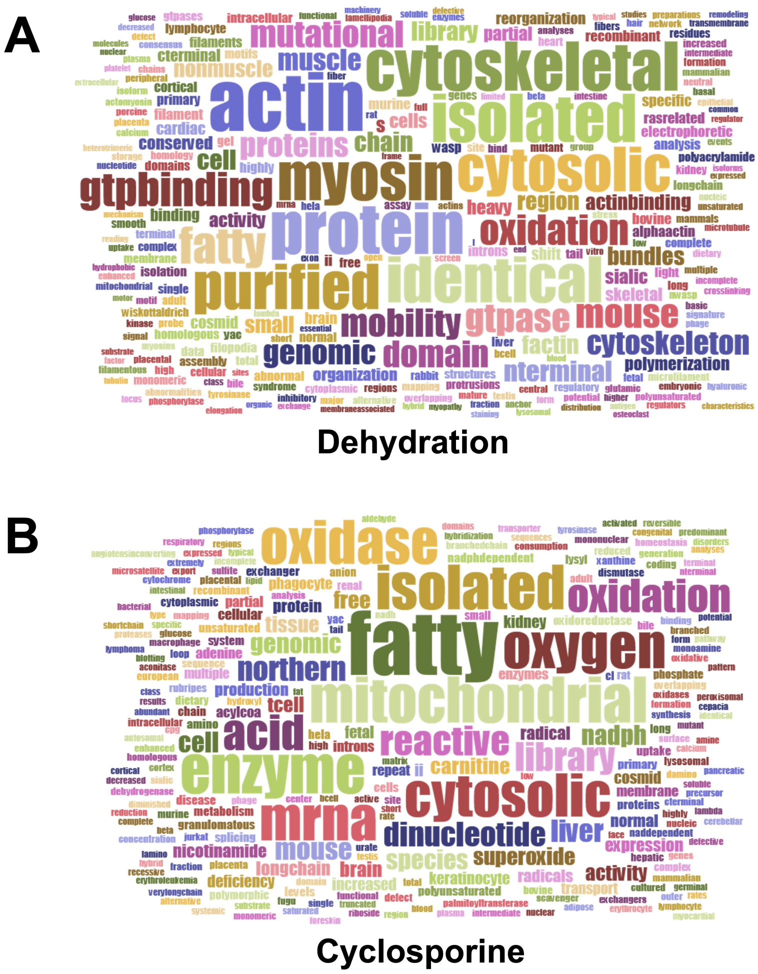


**Figure S5.** ***Textrous!*****-based high-dimensionality wordclouds of noun-phrase outputs of the functional divergence between dehydration (A) and cyclosporine (B) effects.** Using the input DEP lists from Dehydration or Cyclosporine treated animals semantically-associated noun-phrases (from databases assembled from OMIM - https://www.omim.org/; PubMed Central - https://www.ncbi.nlm.nih.gov/pmc/; Jackson Labs Mammalian Phenotypes Database - http://www.informatics.jax.org/phenotypes.shtml) associated with the input proteins were then converted to high-dimensionality word clouds (https://www.jasondavies.com/wordcloud/). The size of the resultant text item is proportional to the word frequency in the noun-phrase output.

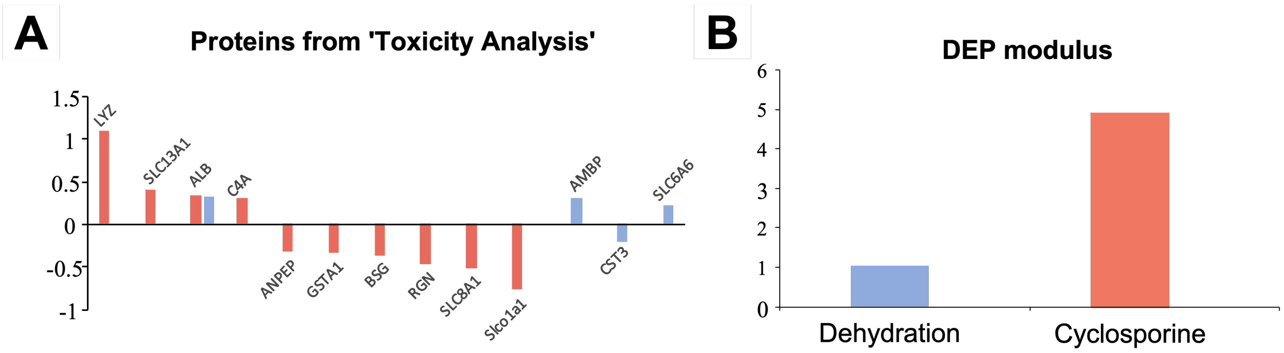


**Figure S6. DEPs invalidating toxicity pathways in the dehydration and cyclosporine cohort**. (**A**) Expression variation of specific proteins from the greater DEP input list from dehydration (blue) and cyclosporine (red) cohorts that populated the Renal Toxicity Analysis. (**B**) Expression values of these proteins demonstrates that the specific cyclosporine DEPs expression variation (red) is to a greater extent associated with renal toxicity-associated DEPs than the dehydration derived DEPs (blue).

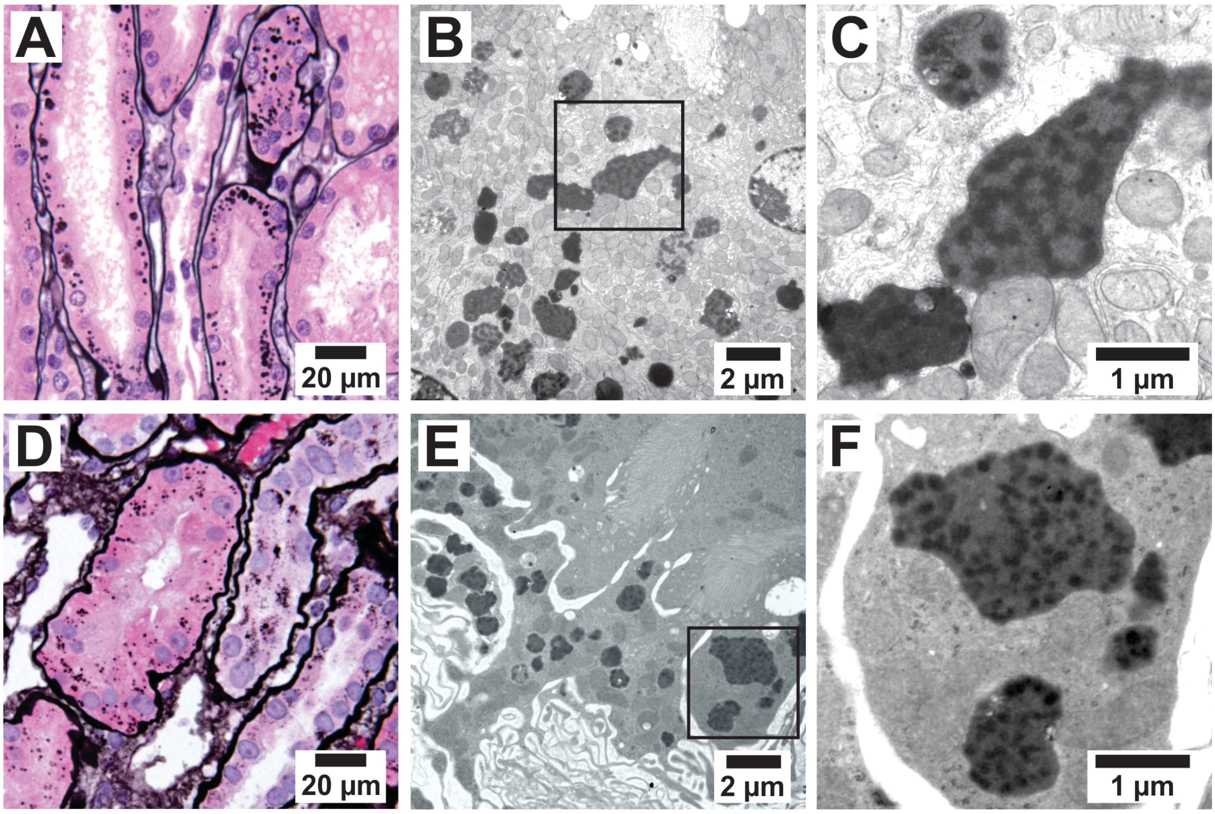


**Figure S7. Renal biopsies of transplant patients after cyclosporine- (A-C) and tacrolimus-treatment (D-F).** PASM stain showing silver-positive lysosomes in proximal tubules on LM (**A**, **D**) and dysmorphic lysosomes containing dark electron-dense aggregates on TEM (**B, C, E, F**), all observed in proximal tubules. Image reproduced from Vervaet et al. who published with Elsevier as an open access article under the CC BY-NC-ND license (http://creativecommons.org/licenses/by-nc-nd/4.0/).
