# Supplementary material for: Chronic dehydration induces injury pathways in rats, but does not mimic histopathology of chronic interstitial nephritis in agricultural communities
Source: Sci Rep. 2023 Oct 23;13:18119. doi: 10.1038/s41598-023-43567-z (PMC10593944; doi:10.1038/s41598-023-43567-z)

Supplementary Tables

Table of Contents

Table S1: Differentially expressed renal proteins in response to dehydration protocols

Table S2: Differentially expressed renal proteins in response to cyclosporine treatment

Table S3: Venn diagram analysis of differentially expressed renal proteins in response to either dehydration or cyclosporine treatment

Table S4: Gene Ontology-based interpretation of dehydration-induced proteomic perturbations

Table S5: Gene Ontology-based interpretation of cyclosporine-induced proteomic perturbations

Table S6: Ingenuity Canonical Signaling Pathway Analysis comparison of renal proteomic responses to dehydration or cyclosporine treatment

Table S7: Overlapping of dehydration- and cyclosporine exposure DEPs with a literature-derived protein list

Table S8: Ingenuity Disease/Bio-Function comparison analysis of renal proteomic responses to dehydration or cyclosporine treatment

Table S9: Ingenuity Toxicity Function analysis of renal proteomic responses to dehydration

Table S10: Ingenuity Toxicity Function analysis of renal proteomic responses to cyclosporine treatment

Table S11: Heatmaps of Gene Symbol-Biomedical Text word correlations using input DEP lists from the dehydration cohort showing associated text matrices revealing a clustering of proteins with biomedical terms.

Table S12: Heatmaps of Gene Symbol-Biomedical Text word correlations using input DEP lists from the cyclosporine cohort showing associated text matrices revealing a clustering of proteins with biomedical terms.

Table S1. Differentially expressed renal proteins in response to dehydration protocols. Proteins that demonstrated a significant (p<0.05) deviation from background expression levels, when comparing samples from dehydrated animals compared to controls are depicted in the table. For each differentially regulated protein the Uniprot accession #, description, official Gene Symbol and the log_2 transformed expression ratio (Dehydration:Control) is given.

Accession #	Protein Description	Gene Symbol	Dehydration:Control (Log2)
Q62669	Globin a1	LOC103694855	0.696260912
A0A0G2K7W6	Uncharacterized protein	RGD1562402	0.626260912
P20760	Ig gamma-2A chain C region	Igg-2a	0.536260912
A0A0G2JSV6	Globin c2	Hba-a2	0.446260912
A0A0G2JSW3	Globin a4	Hbb	0.446260912
P11517	Hemoglobin subunit beta-2	Hbb2	0.436260912
P14046	Alpha-1-inhibitor 3	A1i3	0.326260912
A0A0G2JSH5	Serum albumin	Alb	0.316260912
E9PU64	Scinderin	Scin	0.306260912
Q5XIE6	3-hydroxyisobutyryl-CoA hydrolase, mitochondrial	Hibch	0.306260912
B1WBW2	RWD domain-containing 2B	Rwdd2b	0.306260912
P70583	Deoxyuridine 5'-triphosphate nucleotidohydrolase	Dut	0.306260912
Q63041	Alpha-1-macroglobulin	A1m	0.296260912
B0BNN3	Carbonic anhydrase 1	Ca1	0.296260912
B2GV74	Kinesin light chain 2	Klc2	0.286260912
Q03336	Regucalcin	Rgn	0.286260912
P14668	Annexin A5	Anxa5	0.266260912
D4A833	Mitochondrial ribosomal protein S30	Mrps30	0.266260912
P06686	Sodium/potassium-transporting ATPase subunit alpha-2	Atp1a2	0.266260912
F8WFT7	Anion exchange protein	Slc4a1	0.266260912
P23928	Alpha-crystallin B chain	Cryab	0.266260912
D3ZAS9	DDRGK domain-containing 1	Ddrgk1	0.256260912
D3ZPY8	Aldo-keto reductase family 1, member C13	Akr1c13	0.256260912
P08934	Kininogen-1	Kng1	0.236260912
A0A0G2JW12	Complement component 4A (Rodgers blood group)	C4a	0.236260912
Q63356	Unconventional myosin-Ie	Myo1e	0.236260912
Q9WTQ2	Podocalyxin	Podxl	0.236260912
D3ZAF6	ATP synthase subunit f, mitochondrial	Atp5j2	0.236260912
F1LPQ6	Uncharacterized protein	Ighm	0.236260912
M0R3Z8	RCG28930, isoform CRA_b	Rbm15	0.226260912
Q4V7C6	GMP synthase [glutamine-hydrolyzing]	Gmps	0.226260912
A0A0G2K8V5	C-reactive protein	Crp	0.226260912
P13697	NADP-dependent malic enzyme	Me1	0.226260912
Q01984	Histamine N-methyltransferase	Hnmt	0.226260912
P05964	Protein S100-A6	S100a6	0.216260912
A0A0G2K654	Histone cluster 1 H1 family member c	Hist1h1c	0.216260912

A0A0G2K227	Transporter	Slc6a6	0.216260912
P14841	Cystatin-C	Cst3	-0.213739088
D3ZTR5	RCG21454	Zbed5	-0.213739088
C7E1V1	Inositol 1,4,5-trisphosphate receptor type 3	Itpr3	-0.213739088
P05503	Cytochrome c oxidase subunit 1	Mtco1	-0.213739088
Q5XIP9	Transmembrane protein 43	Tmem43	-0.213739088
Q6AYI2	Kelch domain-containing protein 3	Klhdc3	-0.223739088
F1LTJ5	Uncharacterized protein	F1LTJ5	-0.223739088
Q5XIC2	Evolutionarily conserved signaling intermediate in Toll pathway, mitochondrial	Ecsit	-0.223739088
P53042	Serine/threonine-protein phosphatase 5	Ppp5c	-0.223739088
M0RAP5	SET-binding factor 1	Sbf1	-0.233739088
D4A3E1	Heterogeneous nuclear ribonucleoprotein L-like	Hnrnpll	-0.233739088
A0A0G2K0P5	Thyroid hormone receptor interactor 4	Trip4	-0.233739088
Q03344	ATPase inhibitor, mitochondrial	Atpif1	-0.233739088
B5DFJ4	VPS18 CORVET/HOPS core subunit	Vps18	-0.233739088
Q5M876	N-acyl-aromatic-L-amino acid amidohydrolase (carboxylate-forming)	Acy3	-0.233739088
P02761	Major urinary protein	Mup	-0.233739088
M0R8V0	UHRF1-binding protein 1-like	LOC100910056	-0.243739088
D3ZZ38	Sorting nexin	Snx18	-0.243739088
P18445	60S ribosomal protein L27a	Rpl27a	-0.243739088
P18297	Sepiapterin reductase	Spr	-0.243739088
Q6IN37	GM2 ganglioside activator	Gm2a	-0.253739088
O54772	SWI/SNF-related matrix-associated actin-dependent regulator of chromatin subfamily D member 2	Smarcd2	-0.253739088
F7ES73	Negative regulator of ubiquitin-like proteins 1	Nub1	-0.253739088
A0A0G2K0V3	3'(2'),5'-bisphosphate nucleotidase 1	Bpnt1	-0.253739088
B1PLB1	CD34 antigen (Predicted)	Cd34	-0.253739088
Q3MID4	Carbohydrate kinase-like, isoform CRA_b	Shpk	-0.253739088
D4A914	5'-3' exoribonuclease 2	Xrn2	-0.253739088
Q4KLI7	Splicing factor 3a, subunit 3	Sf3a3	-0.253739088
A0A0G2K8S6	Glucosidase 1	LOC103692171	-0.253739088
Q9WUF4	Vesicle-associated membrane protein 8	Vamp8	-0.253739088
F1LN59	Eukaryotic translation initiation factor 4, gamma 2	Eif4g2	-0.263739088
Q32PZ3	Protein unc-45 homolog A	Unc45a	-0.263739088
G3V928	LDL receptor-related protein 1	Lrp1	-0.263739088
Q66H98	Serum deprivation-response protein	Sdpr	-0.263739088
P97538	Ras-related protein M-Ras	Mras	-0.263739088
P20611	Lysosomal acid phosphatase	Acp2	-0.273739088
F7F350	Synapse associated protein 1, isoform CRA_a	Syap1	-0.273739088
G3V7B3	Chloride channel protein	Clcnkb	-0.273739088
B2RYI2	Signal recognition particle subunit SRP68	Srp68	-0.273739088
F1M3W5	Dmx-like 2	Dmxl2	-0.273739088

D3ZK45	Uncharacterized protein	Epb41l5	-0.283739088
Q5BK74	Transcription elongation factor A (SII), 3	Tcea3	-0.283739088
Q9JLA3	UDP-glucose:glycoprotein glucosyltransferase 1	Uggt1	-0.293739088
D3ZMY7	5'-nucleotidase, cytosolic II	Nt5c2	-0.293739088
A0A0G2K5K7	Inositol polyphosphate-5-phosphatase K	Inpp5k	-0.293739088
O54715	V-type proton ATPase subunit S1	Atp6ap1	-0.293739088
P62870	Elongin-B	Elob	-0.293739088
A0A0G2K695	Myoferlin	Myof	-0.303739088
D3ZCD4	Phosphatidylinositol transfer protein beta isoform	Pitpnb	-0.303739088
D3ZM09	Seryl-tRNA synthetase 2 (Predicted)	Sars2	-0.303739088
Q9R066	Coxsackievirus and adenovirus receptor homolog	Cxadr	-0.323739088
O35567	Bifunctional purine biosynthesis protein PURH	Atic	-0.323739088
D4A031	DEAD (Asp-Glu-Ala-Asp) box polypeptide 42 (Predicted)	Ddx42	-0.323739088
Q64240	Protein AMBP	Ambp	-0.323739088
B1WBY5	DnaJ (Hsp40) homolog, subfamily C, member 11	Dnajc11	-0.333739088
P19234	NADH dehydrogenase [ubiquinone] flavoprotein 2, mitochondrial	Ndufv2	-0.333739088
D4A4H5	Stromal cell derived factor 2 (Predicted), isoform CRA_b	Sdf2	-0.343739088
Q68FV6	Glycosylated lysosomal membrane protein	Glmp	-0.343739088
A0A0G2JZG7	Serine--tRNA ligase, cytoplasmic	Sars	-0.363739088
D3ZGE6	Cortactin, isoform CRA_c	Cttn	-0.373739088
Q498T4	39S ribosomal protein L2, mitochondrial	Mrpl2	-0.383739088
P36506	Dual specificity mitogen-activated protein kinase kinase 2	Map2k2	-0.393739088
P20037	Rano class II histocompatibility antigen, B alpha chain	RT1-Ba	-0.403739088
Q6IMX3	Acetyl-Coenzyme A dehydrogenase, short chain, isoform CRA_a	Acads	-0.423739088
D3ZE63	Uncharacterized protein	LOC679748	-0.423739088
P25236	Selenoprotein P	Selenop	-0.513739088
A0A0G2K394	Low molecular weight phosphotyrosine protein phosphatase	Acp1	-0.583739088
D4A0Y4	Oxidoreductase NAD-binding domain containing 1 (Predicted), isoform CRA_b	Oxnad1	-0.683739088
P59382	Peroxisomal membrane protein 4	Pxmp4	-0.693739088
Q5BKC6	HSPB1-associated protein 1	Hspbap1	-0.723739088
D3ZVK7	Histone H2A	Hist1h2ak	-0.743739088

Table S2. Differentially expressed renal proteins in response to cyclosporine treatment. Proteins that demonstrated a significant ($p<0.05$) deviation from background expression levels, when comparing samples from cyclosporine-treated animals compared to controls are depicted in the table. For each differentially regulated protein the Uniprot accession #, description, official Gene Symbol and the log_2 transformed expression ratio (Cyclosporine:Control) is given.

Accession #	Protein Description	Gene Symbol	Cyclosporine:Control (Log2)
F1M8E9	Lysozyme f2	Lyz2	1.09600388
P08424	Renin	Ren1	0.94600388
Q6AYR1	RCG52996, isoform CRA_a	Tfg	0.76600388
P06302	Prothymosin alpha	Ptma	0.76600388
P08934	Kininogen-1	Kng1	0.74600388
D4A3X0	Uncharacterized protein	Cep192	0.66600388
A0A0G2K1G0	BCL2-associated transcription factor 1	Bclaf1	0.60600388
G3V6Y9	Peptidyl-prolyl cis-trans isomerase G	Ppig	0.56600388
P20760	Ig gamma-2A chain C region	Igg-2a	0.55600388
Q6MGB4	H2-K region expressed gene 4, rat orthologue	Slc39a7	0.54600388
M0R7Y9	Uncharacterized protein	M0R7Y9	0.54600388
P04550	Parathymosin	Ptms	0.53600388
A0A0G2JWK7	Transgelin	Tagln	0.52600388
G3V8R0	Similar to sid2057p	RGD1311703	0.52600388
A0A0G2K6H6	Sodium-dependent phosphate transport protein 1	Slc17a1	0.52600388
Q4KLJ0	High mobility group nucleosomal binding domain 2	LOC100360316	0.51600388
D4ACN6	Collagen type IV alpha 3-binding protein	Col4a3bp	0.51600388
P20411	High affinity immunoglobulin epsilon receptor subunit gamma	Fcer1g	0.50600388
P24268	Cathepsin D	Ctsd	0.49600388
G3V8L1	PYD and CARD domain containing	Pycard	0.49600388
Q91V33	KH domain-containing, RNA-binding, signal transduction-associated protein 1	Khdrbs1	0.48600388
P47875	Cysteine and glycine-rich protein 1	Csrp1	0.48600388
D3ZBN0	Histone H1.5	Hist1h1b	0.45600388
F1M1W1	Acyl-CoA synthetase medium-chain family member 1	Acsm1	0.45600388
Q9Z2Y0	Glycine N-acyltransferase-like protein Keg1	Keg1	0.44600388
Q499V1	Uridine phosphorylase	Upp1	0.42600388
F1M446	Uncharacterized protein	RGD1306148	0.42600388
A0A0G2K2M9	Serine/arginine repetitive matrix 2	Srrm2	0.41600388
D4A8Z3	Ferric-chelate reductase 1	Frrs1	0.41600388
Q6AYI2	Kelch domain-containing protein 3	Klhdc3	0.41600388
Q6IN37	GM2 ganglioside activator	Gm2a	0.41600388

A0A0G2K014	Lymphocyte cytosolic protein 1	Lcp1	0.39600388
Q07782	Solute carrier family 13 member 1	Slc13a1	0.39600388
A0A0G2JSZ5	Protein disulfide-isomerase A6	Pdia6	0.38600388
G3V6P6	RNA binding motif protein 3, isoform CRA_a	Rbm3	0.38600388
F1M853	Ribosome-binding protein 1	Rrbp1	0.38600388
P05964	Protein S100-A6	S100a6	0.37600388
Q9QX67	Death-associated protein 1	Dap	0.37600388
A0A0G2K654	Histone cluster 1 H1 family member c	Hist1h1c	0.36600388
P07150	Annexin A1	Anxa1	0.36600388
A0A0G2JSQ3	Solute carrier family 22 (Organic anion transporter), member 8, isoform CRA_a	Slc22a8	0.36600388
P14668	Annexin A5	Anxa5	0.35600388
Q8K585	High mobility group protein HMG-I/HMG-Y	Hmga1	0.35600388
F1LPS3	Nucleolar and coiled-body phosphoprotein 1	Nolc1	0.35600388
D4A6D9	HCLS1-binding protein 3	Hs1bp3	0.35600388
F1MAC0	Interferon gamma-inducible protein 47	Ifi47	0.35600388
F1LMW7	Myristoylated alanine-rich C-kinase substrate	Marcks	0.35600388
Q9WVJ6	Tissue-type transglutaminase	Tgm2	0.34600388
Q5HZY0	UBX domain-containing protein 4	Ubxn4	0.33600388
P02770	Serum albumin	Alb	0.33600388
D4AEG7	TBC1 domain family, member 13	Tbc1d13	0.33600388
Q6P762	Alpha-mannosidase	Man2b1	0.33600388
Q63042	FAD-linked sulfhydryl oxidase ALR	Gfer	0.33600388
O54772	SWI/SNF-related matrix-associated actin-dependent regulator of chromatin subfamily D member 2	Smarcd2	0.33600388
P07872	Peroxisomal acyl-coenzyme A oxidase 1	Acox1	0.32600388
Q5PQK2	Fused in sarcoma RNA-binding protein	Fus	0.32600388
Q07936	Annexin A2	Anxa2	0.31600388
A0A096MKG5	NAD kinase 2, mitochondrial	Nadk2	0.31600388
A0A0G2JW12	Complement component 4A (Rodgers blood group)	C4a	0.30600388
D4A7U1	Zyxin	Zyx	0.30600388
A0A0G2K9B1	Serine protease inhibitor	LOC299282	0.30600388
F1LR87	Beta-hexosaminidase	Hexb	0.30600388
F7FJQ3	NPC intracellular cholesterol transporter 2	Npc2	0.30600388
Q63797	Proteasome activator complex subunit 1	Psme1	0.29600388
D4A779	Pleckstrin homology-like domain, family B, member 2	Phldb2	0.29600388
G3V8X5	Solute carrier family 5 member 10	Slc5a10	-0.29399612
P80432	Cytochrome c oxidase subunit 7C, mitochondrial	Cox7c	-0.29399612
D3ZE15	NADH dehydrogenase [ubiquinone] 1 alpha subcomplex subunit 13-like	LOC100911483	-0.29399612
P03889	NADH-ubiquinone oxidoreductase chain 1	Mtnd1	-0.30399612
Q5U2X8	Acyl-CoA thioesterase 9	Acot9	-0.30399612
G3V7I0	Peroxiredoxin 3	Prdx3	-0.30399612
Q9WUF4	Vesicle-associated membrane protein 8	Vamp8	-0.30399612

P62870	Elongin-B	Elob	-0.30399612
P46844	Biliverdin reductase A	Blvra	-0.31399612
Q9JJI3	Alpha-2u globulin	LOC259244	-0.31399612
P07379	Phosphoenolpyruvate carboxykinase, cytosolic [GTP]	Pck1	-0.31399612
P23764	Glutathione peroxidase 3	Gpx3	-0.31399612
A0A0G2JVE6	Alanyl (Membrane) aminopeptidase	Anpep	-0.32399612
A0A096MJW3	Uncharacterized protein (Fragment)	A0A096MJW3	-0.32399612
D3ZTX4	Uncharacterized protein	Mgam	-0.32399612
M0RAK3	Solute carrier family 12 member 9	Slc12a9	-0.32399612
A2VD16	Aldo-keto reductase family 1, member C12-like 1	Akr1c12l1	-0.33399612
P04903	Glutathione S-transferase alpha-2	Gsta2	-0.34399612
D3ZVK7	Histone H2A	Hist1h2ak	-0.34399612
D3ZD09	Cytochrome c oxidase subunit	Cox6b1	-0.35399612
P80431	Cytochrome c oxidase subunit 7B, mitochondrial	Cox7b	-0.35399612
Q68FV6	Glycosylated lysosomal membrane protein	Glmp	-0.35399612
D4A0Y4	Oxidoreductase NAD-binding domain containing 1 (Predicted), isoform CRA_b	Oxnad1	-0.35399612
P19234	NADH dehydrogenase [ubiquinone] flavoprotein 2, mitochondrial	Ndufv2	-0.36399612
D3ZDM4	Similar to KIAA1919 protein	RGD1310495	-0.37399612
P26453	Basigin	Bsg	-0.37399612
Q64319	Neutral and basic amino acid transport protein rBAT	Slc3a1	-0.39399612
P51907	Excitatory amino acid transporter 3	Slc1a1	-0.39399612
A0A0G2KA82	Synaptophysin-like 1	Sypl1	-0.39399612
B0BNI6	Similar to solute carrier family 25, member 35, isoform CRA_a	Slc25a35	-0.39399612
Q5RKI7	Solute carrier family 7 member 13	Slc7a13	-0.40399612
Q64611	Cysteine sulfinic acid decarboxylase	Csad	-0.40399612
D3ZPY8	Aldo-keto reductase family 1, member C13	Akr1c13	-0.42399612
P15684	Aminopeptidase N	Anpep	-0.42399612
F1LPQ6	Uncharacterized protein	Ighm	-0.44399612
D3ZE63	Uncharacterized protein	LOC679748	-0.46399612
P59382	Peroxisomal membrane protein 4	Pxmp4	-0.46399612
Q03336	Regucalcin	Rgn	-0.47399612
M0R4N4	Dehydrogenase/reductase (SDR family) member 7-like 1	Dhrs7l1	-0.48399612
P20816	Cytochrome P450 4A2	Cyp4a2	-0.49399612
Q01728	Sodium/calcium exchanger 1	Slc8a1	-0.52399612
Q5BKC6	HSPB1-associated protein 1	Hspbap1	-0.56399612
Q01984	Histamine N-methyltransferase	Hnmt	-0.58399612
P07171	Calbindin	Calb1	-0.67399612
Q6AYQ9	Peptidyl-prolyl cis-trans isomerase	Ppic	-0.72399612
P46720	Solute carrier organic anion transporter family member 1A1	Slco1a1	-0.76399612
A0A0G2JSH2	3-hydroxybutyrate dehydrogenase, type 1, isoform	Bdh1	-0.77399612

	CRA_a		
P02761	Major urinary protein	Mup	-1.08399612

Table S3. Venn diagram analysis of differentially expressed renal proteins in response to either dehydration or cyclosporine treatment. For each significantly-regulated protein the respective $\log_2$ transform of the expression change (positive for upregulation versus control rats, negative for downregulation versus control rats) and the official Gene Symbol are given.

Gene Symbol	Dehydration:Control	Cyclosporine:Control
Igg-2a	0.536260912	0.55600388
Alb	0.316260912	0.33600388
Anxa5	0.266260912	0.35600388
Kng1	0.236260912	0.74600388
C4a	0.236260912	0.30600388
S100a6	0.216260912	0.37600388
Hist1h1c	0.216260912	0.36600388
Mup	-0.233739088	-1.08399612
Vamp8	-0.253739088	-0.30399612
Elob	-0.293739088	-0.30399612
Ndufv2	-0.333739088	-0.36399612
Glmp	-0.343739088	-0.35399612
LOC679748	-0.423739088	-0.46399612
Oxnad1	-0.683739088	-0.35399612
Pxmp4	-0.693739088	-0.46399612
Hspbap1	-0.723739088	-0.56399612
Hist1h2ak	-0.743739088	-0.34399612
Rgn	0.286260912	-0.47399612
Akr1c13	0.256260912	-0.42399612
Ighm	0.236260912	-0.44399612
Hnmt	0.226260912	-0.58399612
Klhdc3	-0.223739088	0.41600388
Gm2a	-0.253739088	0.41600388
Smarcd2	-0.253739088	0.33600388
LOC103694855	0.696260912	
RGD1562402	0.626260912	
Hba-a2	0.446260912	
Hbb	0.446260912	
Hbb2	0.436260912	
A1i3	0.326260912	
Scin	0.306260912	
Hibch	0.306260912	
Rwdd2b	0.306260912	
Dut	0.306260912	
A1m	0.296260912	
Ca1	0.296260912	
Klc2	0.286260912	

Mrps30	0.266260912	
Atp1a2	0.266260912	
Slc4a1	0.266260912	
Cryab	0.266260912	
Ddrgk1	0.256260912	
Myo1e	0.236260912	
Podxl	0.236260912	
Atp5j2	0.236260912	
Rbm15	0.226260912	
Gmps	0.226260912	
Crp	0.226260912	
Me1	0.226260912	
Slc6a6	0.216260912	
Cst3	-0.213739088	
Zbed5	-0.213739088	
Itpr3	-0.213739088	
Mtco1	-0.213739088	
Tmem43	-0.213739088	
F1LTJ5	-0.223739088	
Ecsit	-0.223739088	
Ppp5c	-0.223739088	
Sbf1	-0.233739088	
Hnrnpll	-0.233739088	
Trip4	-0.233739088	
Atpif1	-0.233739088	
Vps18	-0.233739088	
Acy3	-0.233739088	
LOC100910056	-0.243739088	
Snx18	-0.243739088	
Rpl27a	-0.243739088	
Spr	-0.243739088	
Nub1	-0.253739088	
Bpnt1	-0.253739088	
Cd34	-0.253739088	
Shpk	-0.253739088	
Xrn2	-0.253739088	
Sf3a3	-0.253739088	
LOC103692171	-0.253739088	
Eif4g2	-0.263739088	
Unc45a	-0.263739088	
Lrp1	-0.263739088	
Sdpr	-0.263739088	
Mras	-0.263739088	
Acp2	-0.273739088	

Syap1	-0.273739088	
Clcnkb	-0.273739088	
Srp68	-0.273739088	
Dmxl2	-0.273739088	
Epb41l5	-0.283739088	
Tcea3	-0.283739088	
Uggt1	-0.293739088	
Nt5c2	-0.293739088	
Inpp5k	-0.293739088	
Atp6ap1	-0.293739088	
Myof	-0.303739088	
Pitpnb	-0.303739088	
Sars2	-0.303739088	
Cxadr	-0.323739088	
Atic	-0.323739088	
Ddx42	-0.323739088	
Ambp	-0.323739088	
Dnajc11	-0.333739088	
Sdf2	-0.343739088	
Sars	-0.363739088	
Cttn	-0.373739088	
Mrpl2	-0.383739088	
Map2k2	-0.393739088	
RT1-Ba	-0.403739088	
Acads	-0.423739088	
Selenop	-0.513739088	
Acp1	-0.583739088	
Lyz2		1.09600388
Ren1		0.94600388
Tfg		0.76600388
Ptma		0.76600388
Cep192		0.66600388
Bclaf1		0.60600388
Ppig		0.56600388
Slc39a7		0.54600388
M0R7Y9		0.54600388
Ptms		0.53600388
Tagln		0.52600388
RGD1311703		0.52600388
Slc17a1		0.52600388
LOC100360316		0.51600388
Col4a3bp		0.51600388
Fcer1g		0.50600388
Ctsd		0.49600388

Pycard		0.49600388
Khdrbs1		0.48600388
Csrp1		0.48600388
Hist1h1b		0.45600388
Acsm1		0.45600388
Keg1		0.44600388
Upp1		0.42600388
RGD1306148		0.42600388
Srrm2		0.41600388
Frrs1		0.41600388
Lcp1		0.39600388
Slc13a1		0.39600388
Pdia6		0.38600388
Rbm3		0.38600388
Rrbp1		0.38600388
Dap		0.37600388
Anxa1		0.36600388
Slc22a8		0.36600388
Hmga1		0.35600388
Nolc1		0.35600388
Hs1bp3		0.35600388
Ifi47		0.35600388
Marcks		0.35600388
Tgm2		0.34600388
Ubxn4		0.33600388
Tbc1d13		0.33600388
Man2b1		0.33600388
Gfer		0.33600388
Acox1		0.32600388
Fus		0.32600388
Anxa2		0.31600388
Nadk2		0.31600388
Zyx		0.30600388
LOC299282		0.30600388
Hexb		0.30600388
Npc2		0.30600388
Psme1		0.29600388
Phldb2		0.29600388
Slc5a10		-0.29399612
Cox7c		-0.29399612
LOC100911483		-0.29399612
Mtnd1		-0.30399612
Acot9		-0.30399612
Prdx3		-0.30399612

Blvra		-0.31399612
LOC259244		-0.31399612
Pck1		-0.31399612
Gpx3		-0.31399612
Anpep		-0.32399612
A0A096MJW3		-0.32399612
Mgam		-0.32399612
Slc12a9		-0.32399612
Akr1c12l1		-0.33399612
Gsta2		-0.34399612
Cox6b1		-0.35399612
Cox7b		-0.35399612
RGD1310495		-0.37399612
Bsg		-0.37399612
Slc3a1		-0.39399612
Slc1a1		-0.39399612
Sypl1		-0.39399612
Slc25a35		-0.39399612
Slc7a13		-0.40399612
Csad		-0.40399612
Dhrs7l1		-0.48399612
Cyp4a2		-0.49399612
Slc8a1		-0.52399612
Calb1		-0.67399612
Ppic		-0.72399612
Slco1a1		-0.76399612
Bdh1		-0.77399612

Table S4. Gene Ontology-based interpretation of dehydration-induced proteomic perturbations. Gene Ontology terms that demonstrated a significant (p<0.05) enrichment using data from dehydrated animals compared to controls are indicated. For each differentially regulated Gene Ontology (GO) term the process name, GO term ID, enrichment p value (P-value) as well as the proteins form the input data set that populate the specific GO term are represented.

GO Biological Process Term	GO term ID	P-value	Proteins
organic anion transport	GO:0015711	6.01E-05	SLC6A6;CA1;PITPNB;ALB;HBB;SLC4A1
purine ribonucleoside monophosphate metabolic process	GO:0009167	6.33E-05	ATIC;GMPS;ATP1A2;NT5C2
endocytosis	GO:0006897	5.79E-04	MYO1E;SNX18;AMBP;CTTN;LRP1;ALB;HBB
pteridine-containing compound biosynthetic process	GO:0042559	5.96E-04	SPR;ATPIF1
glomerular visceral epithelial cell development	GO:0072015	7.92E-04	MYO1E;PODXL
glomerular epithelial cell development	GO:0072310	7.92E-04	MYO1E;PODXL
glomerular filtration	GO:0003094	0.001263836	MYO1E;CD34
regulated exocytosis	GO:0045055	0.001292202	SCIN;SELENOP;ALB;ANXA5;KNG1
purine ribonucleoside monophosphate biosynthetic process	GO:0009168	0.001308246	ATIC;GMPS;ATP5J2
bicarbonate transport	GO:0015701	0.001508262	CA1;HBB;SLC4A1
renal filtration	GO:0097205	0.00153925	MYO1E;CD34
purine nucleoside monophosphate biosynthetic process	GO:0009127	0.001840599	ATIC;GMPS
positive regulation of transport	GO:0051050	0.001842571	SCIN;LRP1;INPP5K
negative regulation of hemostasis	GO:1900047	0.0021676	CD34;KNG1
glomerular visceral epithelial cell differentiation	GO:0072112	0.0021676	MYO1E;PODXL
ER-associated misfolded protein catabolic process	GO:0071712	0.0021676	SDF2;UGGT1
ribonucleoside monophosphate biosynthetic process	GO:0009156	0.002519972	ATIC;GMPS
regulation of extracellular matrix disassembly	GO:0010715	0.002519972	CST3;LRP1
negative regulation of coagulation	GO:0050819	0.002897438	CD34;KNG1
receptor-mediated endocytosis	GO:0006898	0.003633962	AMBP;CTTN;LRP1;ALB;HBB
NADP metabolic process	GO:0006739	0.004652742	SHPK;ME1
negative regulation of calcium ion transport	GO:0051926	0.004652742	INPP5K;ATP1A2
platelet degranulation	GO:0002576	0.00472532	SELENOP;ALB;ANXA5;KNG1
vesicle organization	GO:0016050	0.00513798	VAMP8;VPS18;SNX18;CD34
cellular response to misfolded protein	GO:0071218	0.005673876	SDF2;UGGT1

purine ribonucleotide metabolic process	GO:0009150	0.005709297	ATP1A2;BPNT1;NT5C2
regulation of cardiac conduction	GO:1903779	0.005949632	CXADR;ITPR3;ATP1A2
positive regulation of proteolysis involved in cellular protein catabolic process	GO:1903052	0.006219384	DDRGK1;ATPIF1
cellular response to epidermal growth factor stimulus	GO:0071364	0.008628278	SYAP1;INPP5K
negative regulation of wound healing	GO:0061045	0.008628278	CD34;KNG1
misfolded or incompletely synthesized protein catabolic process	GO:0006515	0.008628278	SDF2;UGGT1
hydrogen ion transmembrane transport	GO:1902600	0.010666021	ATP6AP1;ATP5J2
coenzyme biosynthetic process	GO:0009108	0.011387949	SPR;ATPIF1
regulation of blood coagulation	GO:0030193	0.012130806	CD34;KNG1
proton transport	GO:0015992	0.01289435	ATP6AP1;ATP5J2
post-translational protein modification	GO:0043687	0.013123909	CST3;C4A;NUB1;ALB;ELOB;KNG1
regulation of nitric oxide biosynthetic process	GO:0045428	0.013678342	HBB;CD34
regulation of cell death	GO:0010941	0.015644367	LRP1;HBB;CRYAB
positive regulation of RNA metabolic process	GO:0051254	0.016150616	MAP2K2;HNRNPLL
negative regulation of blood coagulation	GO:0030195	0.017014025	CD34;KNG1
negative regulation of transport	GO:0051051	0.020658095	INPP5K;CRYAB
regulation of ATPase activity	GO:0043462	0.020658095	ATPIF1;RGN
tRNA aminoacylation	GO:0043039	0.020658095	SARS2;SARS
negative regulation of proteolysis	GO:0045861	0.021615589	CST3;KNG1
cellular response to insulin stimulus	GO:0032869	0.022300631	ATP6AP1;SYAP1;INPP5K
positive regulation of multicellular organismal process	GO:0051240	0.024379176	VAMP8;LRP1;INPP5K;CD34
carboxylic acid biosynthetic process	GO:0046394	0.024596058	ATPIF1;RGN
tRNA aminoacylation for protein translation	GO:0006418	0.026670848	SARS2;SARS
protein heterooligomerization	GO:0051291	0.029910477	HBB;ITPR3
dephosphorylation	GO:0016311	0.030274762	PPP5C;INPP5K;SBF1
negative regulation of macromolecule metabolic process	GO:0010605	0.030274762	MAP2K2;DDRGK1;CD34
protein oligomerization	GO:0051259	0.030580844	ATPIF1;HBB;ITPR3;CRYAB
fatty acid oxidation	GO:0019395	0.031023499	ACADS;HIBCH
fatty acid beta-oxidation	GO:0006635	0.031023499	ACADS;HIBCH

positive regulation of actin filament polymerization	GO:0030838	0.032152748	SCIN;CTTN
cytoplasmic translation	GO:0002181	0.035635833	MRPL2;RPL27A
translation	GO:0006412	0.037635005	MRPL2;SARS2;RPL27A;SARS
negative regulation of cellular catabolic process	GO:0031330	0.038035332	CST3;EIF4G2
ATP metabolic process	GO:0046034	0.038035332	ATP1A2;ATP5J2
positive regulation of cell death	GO:0010942	0.039257722	LRP1;HBB
positive regulation of protein binding	GO:0032092	0.043013126	LRP1;SYAP1
actin cytoskeleton reorganization	GO:0031532	0.044293703	CXADR;CTTN
negative regulation of peptidase activity	GO:0010466	0.045588353	CST3;KNG1
regulation of proteolysis	GO:0030162	0.04689689	CST3;KNG1
cellular protein metabolic process	GO:0044267	0.047789743	CST3;C4A;RPL27A;ALB;SARS;KNG1
NADH dehydrogenase complex assembly	GO:0010257	0.04821913	ECSIT;NDUFV2
mitochondrial respiratory chain complex I biogenesis	GO:0097031	0.04821913	ECSIT;NDUFV2
mitochondrial respiratory chain complex I assembly	GO:0032981	0.04821913	ECSIT;NDUFV2
cellular macromolecule biosynthetic process	GO:0034645	0.049360676	PPP5C;DUT;RPL27A;SARS;EIF4G2
negative regulation of cell adhesion	GO:0007162	0.049554889	PODXL;KNG1
fatty acid catabolic process	GO:0009062	0.049554889	ACADS;HIBCH

Table S5. Gene Ontology-based interpretation of cyclosporine-induced proteomic perturbations. Gene Ontology terms that demonstrated a significant ($p<0.05$) enrichment using data from cyclosporine-treated animals compared to controls are indicated. For each differentially regulated Gene Ontology (GO) term the process name, GO term ID, enrichment p value (P-value) as well as the proteins form the input data set that populate the specific GO term are represented.

GO Biological Process Term	GO term ID	P-value	Proteins
neutrophil degranulation	GO:0043312	8.05E-05	PYCARD;VAMP8;MGAM;FCER1G;GM2A;ANXA2;NPC2;ANPEP;HEXB;MAN2B1;CTSD
neutrophil activation involved in immune response	GO:0002283	8.66E-05	PYCARD;VAMP8;MGAM;FCER1G;GM2A;ANXA2;NPC2;ANPEP;HEXB;MAN2B1;CTSD
neutrophil mediated immunity	GO:0002446	9.32E-05	PYCARD;VAMP8;MGAM;FCER1G;GM2A;ANXA2;NPC2;ANPEP;HEXB;MAN2B1;CTSD
mitochondrial electron transport, cytochrome c to oxygen	GO:0006123	2.11E-04	COX7B;COX7C;COX6B1
sodium ion transport	GO:0006814	3.35E-04	SLC13A1;SLC5A10;SLC17A1;SLC8A1
positive regulation of vesicle fusion	GO:0031340	4.59E-04	ANXA1;ANXA2
sodium ion transmembrane transport	GO:0035725	0.001156293	SLC13A1;SLC17A1;SLC8A1
mitochondrial ATP synthesis coupled electron transport	GO:0042775	0.00139034	COX7B;NDUFV2;COX7C;COX6B1
positive regulation of phagocytosis	GO:0050766	0.001452486	PYCARD;C4A;FCER1G
myeloid cell activation involved in immune response	GO:0002275	0.001653947	PYCARD;FCER1G
respiratory electron transport chain	GO:0022904	0.002005672	COX7B;NDUFV2;COX7C;COX6B1
interleukin-12-mediated signaling pathway	GO:0035722	0.002317069	ANXA2;GSTA2;LCP1
cellular response to interleukin-12	GO:0071349	0.002317069	ANXA2;GSTA2;LCP1
hydrogen peroxide catabolic process	GO:0042744	0.00270667	PRDX3;GPX3
intracellular lipid transport	GO:0032365	0.003111689	COL4A3BP;NPC2
cellular response to glucocorticoid stimulus	GO:0071385	0.004001047	ANXA1;PCK1

anion transport	GO:0006820	0.004015273	SLC13A1;SLC17A1;SLC22A8
nucleosome disassembly	GO:0006337	0.004994149	SMARCD2;HMGA1
chromatin disassembly	GO:0031498	0.004994149	SMARCD2;HMGA1
regulation of vesicle fusion	GO:0031338	0.005086139	ANXA1;ANXA2;TBC1D13
organic anion transport	GO:0015711	0.005372972	NPC2;ALB;SLC22A8;SLC17A1
hydrogen peroxide metabolic process	GO:0042743	0.005528855	PRDX3;GPX3
protein-DNA complex disassembly	GO:0032986	0.006088606	SMARCD2;HMGA1
phagocytosis	GO:0006909	0.007213714	IGHM;ANXA1;PDIA6;TGM2
regulation of interleukin-8 secretion	GO:2000482	0.007282073	PYCARD;ANXA1
inorganic cation transmembrane transport	GO:0098662	0.008172624	SLC13A1;SLC39A7;SLC17A1;SLC8A1
positive regulation of endopeptidase activity	GO:0010950	0.008572242	PYCARD;PSME1
nitrogen compound transport	GO:0071705	0.008617294	COL4A3BP;SLC3A1;SLC17A1
negative regulation of wound healing	GO:0061045	0.009252879	PHLDB2;KNG1
L-amino acid transport	GO:0015807	0.009252879	SLC1A1;SLC3A1
antigen processing and presentation of exogenous peptide antigen via MHC class I	GO:0042590	0.009930695	VAMP8;FCER1G;PSME1
apoptotic cell clearance	GO:0043277	0.009956843	PDIA6;TGM2
T cell activation involved in immune response	GO:0002286	0.009956843	FCER1G;LCP1
regulation of blood coagulation	GO:0030193	0.013000453	FCER1G;KNG1
negative regulation of secretion by cell	GO:1903531	0.013000453	VAMP8;ANXA1

liver development	GO:0001889	0.013816933	PCK1;GFER
post-translational protein modification	GO:0043687	0.015472269	C4A;ALB;PSME1;ELOB;PDIA6;KNG1
positive regulation of organelle organization	GO:0010638	0.015514716	ANXA1;ANXA2
epithelial cell differentiation	GO:0030855	0.017225799	TAGLN;GSTA2;PCK1
sodium-independent organic anion transport	GO:0043252	0.019162254	ALB;SLC22A8
protein peptidyl-prolyl isomerization	GO:0000413	0.019162254	PPIG;PPIC
cellular protein metabolic process	GO:0044267	0.019363039	C4A;CALB1;ALB;MAN2B1;RRBP1;PDIA6;KNG1
T cell differentiation	GO:0030217	0.020125134	ANXA1;FCER1G
regulation of cellular amide metabolic process	GO:0034248	0.020125134	RBM3;NOLC1
acyl-CoA metabolic process	GO:0006637	0.021107908	ACOT9;ACSM1
peptidyl-proline modification	GO:0018208	0.022110326	PPIG;PPIC
regulation of phagocytosis	GO:0050764	0.023132142	PYCARD;FCER1G
monocarboxylic acid metabolic process	GO:0032787	0.02337909	ACSM1;BSG;PCK1
positive regulation of transport	GO:0051050	0.026311541	ANXA1;ANXA2
inorganic anion transport	GO:0015698	0.026311541	SLC13A1;SLC17A1
posttranscriptional regulation of gene expression	GO:0010608	0.026311541	RBM3;NOLC1
hexose metabolic process	GO:0019318	0.027408523	MAN2B1;PCK1
metal ion homeostasis	GO:0055065	0.027408523	SLC8A1;SLC12A9
sphingolipid metabolic process	GO:0006665	0.028060381	COL4A3BP;GM2A;HEXB

pyruvate metabolic process	GO:0006090	0.028523701	BSG;PCK1
regulation of intrinsic apoptotic signaling pathway	GO:2001242	0.029656841	PYCARD;BCLAF1
amino acid transport	GO:0006865	0.03080771	SLC7A13;SLC3A1
regulation of cellular protein metabolic process	GO:0032268	0.033161716	RBM3;NOLC1
fatty acid oxidation	GO:0019395	0.033161716	ACOX1;ACSM1
platelet degranulation	GO:0002576	0.033203016	ALB;ANXA5;KNG1
coenzyme metabolic process	GO:0006732	0.034364399	ACOT9;ACSM1
glycosphingolipid metabolic process	GO:0006687	0.038072481	GM2A;HEXB
positive regulation of response to stimulus	GO:0048584	0.038072481	BCLAF1;HMGA1
regulation of apoptotic process	GO:0042981	0.039663881	PYCARD;PRDX3;BCLAF1;ANXA1;ALB;ANXA5;HS1BP3;KNG1;TGM2
regulation of secretion by cell	GO:1903530	0.040625696	VAMP8;ANXA1
granulocyte chemotaxis	GO:0071621	0.040625696	ANXA1;FCER1G
organonitrogen compound catabolic process	GO:1901565	0.040625696	ANPEP;CTSD
IRE1-mediated unfolded protein response	GO:0036498	0.041926002	KLHDC3;PDIA6
chloride transmembrane transport	GO:1902476	0.041926002	SLC1A1;SLC12A9
regulation of cytosolic calcium ion concentration	GO:0051480	0.042530658	CALB1;SLC8A1;KNG1
sterol homeostasis	GO:0055092	0.043241822	NPC2;ACSM1
cholesterol homeostasis	GO:0042632	0.043241822	NPC2;ACSM1
positive regulation of T cell proliferation	GO:0042102	0.047280264	PYCARD;ANXA1

Table S6. Ingenuity Canonical Signaling Pathway Analysis comparison of renal proteomic responses to dehydration or cyclosporine treatment. For each defined pathway (n>2 proteins per pathway) the negative $\log_{10}$ of the enrichment p value is given. Statistically significant pathway enrichment is observed from a $\log_{10}$ transformed p value of >1.3.

Canonical Signaling Pathways	dehydration ($-\log_{10}$ p value)	cyclosporine ($-\log_{10}$ p value)
Acute Phase Response Signaling	5.21025649	1.416069508
Chondroitin Sulfate Degradation (Metazoa)	1.213230265	2.693091184
Dermatan Sulfate Degradation (Metazoa)	1.187739513	2.639923278
Mitochondrial Dysfunction	0.307983671	2.226059687
Oxidative Phosphorylation	0.455621228	1.958081763
Glutathione Redox Reactions I	0	2.340870876
Glutathione-mediated Detoxification	0	2.095781672
Taurine Biosynthesis	0	2.075300887
Acyl-CoA Hydrolysis	0	1.306174917
Apelin Adipocyte Signaling Pathway	0	1.347961555
Ketogenesis	0	1.383555939
Ketolysis	0	1.383555939
Thyroid Hormone Biosynthesis	0	1.900114873
Heme Degradation	0	1.776080778
Aryl Hydrocarbon Receptor Signaling	0	1.653602622
Unfolded protein response	0	1.632046506
Glutamate Receptor Signaling	0	1.617708867
NAD Phosphorylation and Dephosphorylation	2.934643497	1.272312016
Purine Nucleotides De Novo Biosynthesis II	3.0841511	0
NAD Salvage Pathway II	3.842878133	0
Selenocysteine Biosynthesis II (Archaea and Eukaryotes)	3.642856116	0
LXR/RXR Activation	1.914958228	1.836442045
Calcium Transport I	1.412310092	1.383555939
Glucose and Glucose-1-phosphate Degradation	1.371758746	1.343063668
FXR/RXR Activation	1.867847524	1.00458504
Clathrin-mediated Endocytosis Signaling	1.31465597	0.663584224
Calcium-induced T Lymphocyte Apoptosis	1.553778414	0.613503175
Role of NFAT in Regulation of the Immune Response	2.153634633	0.254158085
α-Adrenergic Signaling	2.227024973	0.487746813
IL-4 Signaling	2.214160445	0.483952035
CD28 Signaling in T Helper Cells	1.814047652	0.36836186
Fc Epsilon RI Signaling	1.877097794	0.38622327
Natural Killer Cell Signaling	1.849595214	0.378413731
D-myo-inositol (1,4,5)-trisphosphate Degradation	2.647601788	0
PDGF Signaling	2.188879623	0
IL-17 Signaling	2.253218742	0
Non-Small Cell Lung Cancer Signaling	2.364729419	0
Aldosterone Signaling in Epithelial Cells	2.356736838	0
Superpathway of D-myo-inositol (1,4,5)-trisphosphate Metabolism	2.398039579	0
GDNF Family Ligand-Receptor Interactions	2.37949331	0
Cholecystokinin/Gastrin-mediated Signaling	2.059546216	0
tRNA Charging	1.985717667	0
Oncostatin M Signaling	1.964544462	0
EIF2 Signaling	1.905434152	0
Inosine-5'-phosphate Biosynthesis II	1.929284759	0

Tetrahydrobiopterin Biosynthesis I	1.929284759	0
Tetrahydrobiopterin Biosynthesis II	1.929284759	0
IL-6 Signaling	1.796732956	0
UVC-Induced MAPK Signaling	1.796145603	0
Iron homeostasis signaling pathway	1.771309103	0
CCR3 Signaling in Eosinophils	1.779712082	0
PI3K Signaling in B Lymphocytes	1.779712082	0
Synaptic Long Term Potentiation	1.867847524	0
PTEN Signaling	1.886432598	0
PI3K/AKT Signaling	1.831665239	0
fMLP Signaling in Neutrophils	1.840590567	0
Renin-Angiotensin Signaling	1.849595214	0
Thyroid Cancer Signaling	1.848310929	0
NRF2-mediated Oxidative Stress Response	1.351314379	0.242735748
Cancer Drug Resistance By Drug Efflux	1.701420805	0
Insulin Receptor Signaling	1.698724324	0
Melanoma Signaling	1.617278519	0
Regulation of eIF4 and p70S6K Signaling	1.574829684	0
Gαq Signaling	1.588600801	0
Glioblastoma Multiforme Signaling	1.541256436	0
Thrombopoietin Signaling	1.495347034	0
Endometrial Cancer Signaling	1.506663421	0
IL-2 Signaling	1.506663421	0
CNTF Signaling	1.518159341	0
GNRH Signaling	1.521675125	0
CXCR4 Signaling	1.521675125	0
Urate Biosynthesis/Inosine 5'-phosphate Degradation	1.300889182	0
Adrenomedullin signaling pathway	1.351314379	0
Gap Junction Signaling	1.345982023	0
Regulation of IL-2 Expression in Activated and Anergic T Lymphocytes	1.344138596	0
Renal Cell Carcinoma Signaling	1.326228625	0
Erythropoietin Signaling	1.335125727	0
Guanosine Nucleotides Degradation III	1.334811	0
JAK/Stat Signaling	1.317444649	0
Prolactin Signaling	1.317444649	0
IL-3 Signaling	1.317444649	0
Synaptic Long Term Depression	1.465298981	0
Agrin Interactions at Neuromuscular Junction	1.451778556	0
ErbB2-ErbB3 Signaling	1.451778556	0
Neurotrophin/TRK Signaling	1.371899613	0
Antiproliferative Role of Somatostatin Receptor 2	1.391038579	0
B Cell Receptor Signaling	1.384006496	0
IL-15 Signaling	1.38140387	0
BMP signaling pathway	1.38140387	0
Chemokine Signaling	1.430949256	0
Role of MAPK Signaling in the Pathogenesis of Influenza	1.420758756	0
ErbB4 Signaling	1.420758756	0
GM-CSF Signaling	1.410712562	0
SPINK1 General Cancer Pathway	1.410712562	0

Table S7. Overlapping of dehydration- and cyclosporine exposure DEPs with a literature-derived protein list associated with 'dehydration'; 'cyclosporine' 'lysosome'; 'lysosomal storage disease'; 'fibrosis' 'senescence'; and 'mitochondria' compared to a randomly appeared protein list (n=112). Significance is asserted as follows P ≤ 0.01(**); P ≤ 0.001(***).

		Overlapping with signature list	
	Benchmarked associated proteins	Cyclosporine	Dehydration
Dehydration	603	3	11(**)
Cyclosporine	305	8(***)	3
Lysosome	1668	20(***)	14(**)
Lysosomal Storage Disease	654	11(***)	5
Fibrosis	1314	16(***)	7
Senescence	500	10(***)	4(**)
Mitochondria	2642	27(***)	20(**)

Table S8. Ingenuity Disease/Bio-Function comparison analysis of renal proteomic responses to dehydration or cyclosporine treatment. For each IPA-defined Disease/Bio-Function (n>2 proteins per pathway, p<0.05 for enrichment) the predicted Disease/Bio-Function z-score is given. Functions that are considered to be activated are given a positive z score while those considered to be inhibited are given a negative z score.

Disease/Bio-Function	dehydration IPA z score	cyclosporine IPA z score
Apoptosis	0.349	2.538
Cell death	0.262	2.215
Necrosis	0	2.012
Apoptosis of tumor cell lines	0	1.673
Synthesis of reactive oxygen species	-0.095	1.57
Phagocytosis of cells	0	1.519
Engulfment of cells	0	1.392
Cell death of myeloid cells	0	1.161
Proliferation of immune cells	0	1.109
Cell death of phagocytes	0	1.024
Fatty acid metabolism	0	-0.034
Glucose metabolism disorder	0	-0.069
Synthesis of eicosanoid	0	-0.318
Concentration of lipid	0	-0.348
Uptake of D-hexose	0	-0.447
Synthesis of lipid	0	-0.588
Synthesis of fatty acid	0	-0.607
Quantity of steroid	0	-0.895
Disorder of lipid metabolism	0	-1.131
Accumulation of lipid	0	-2.181

Table S9. Ingenuity Toxicity Function analysis of renal proteomic responses to dehydration. For each IPA-defined Toxicity Function (n>2 proteins per pathway) the negative log_{10} of the enrichment p value is given. Statistically significant pathway enrichment is observed from a log_{10} transformed p value of >1.3.

Toxicity Function	negative log_{10} p value
Stage II acute myocardial infarction	4.815308569
Liver cancer	3.348721986
Renal Damage	2.850780887
Nephrosis	2.698970004
Injury of kidney	2.552841969
Liver carcinoma	2.41453927
Proliferation of liver cancer cells	2.296708622
Apoptosis of tubular cells	1.962573502
Apoptosis of cardiomyocytes	1.879426069
Left ventricular dilation	1.812479279

Table S10. Ingenuity Toxicity Function analysis of renal proteomic responses to cyclosporine treatment. For each IPA-defined Toxicity Function (n>2 proteins per pathway) the negative log_{10} of the enrichment p value is given. Statistically significant pathway enrichment is observed from a log_{10} transformed p value of >1.3.

Toxicity Function	negative log_{10} p value
Proximal tubular toxicity	5.57024772
Microvesicular hepatic steatosis	3.863279433
Cholestasis	3.038578906
Acute kidney injury	2.76700389
Damage of kidney	2.754487332
Hyperbilirubinemia	2.455931956
Dysfunction of kidney	2.341035157
Proliferation of liver cancer cells	2.240332155
Hepatocellular carcinoma	1.804100348
Hepatitis B virus-related hepatocellular carcinoma	1.793174124

Table S11. Heatmaps of Gene Symbol-Biomedical Text word correlations using input DEP lists from the dehydration cohort showing associated text matrices revealing a clustering of proteins with biomedical terms.

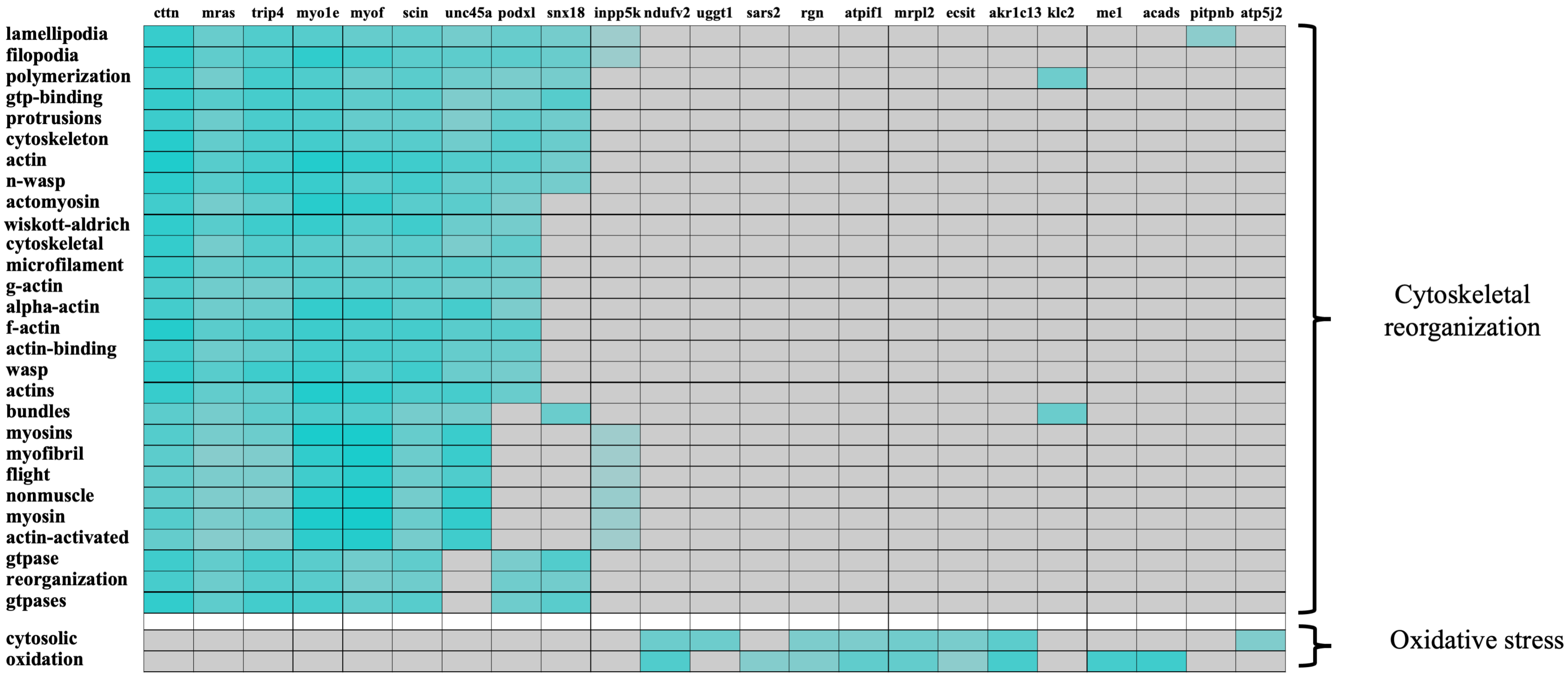

Table S12. Heatmaps of Gene Symbol-Biomedical Text word correlations using input DEP lists from the cyclosporine cohort showing associated text matrices revealing a clustering of proteins with biomedical terms.

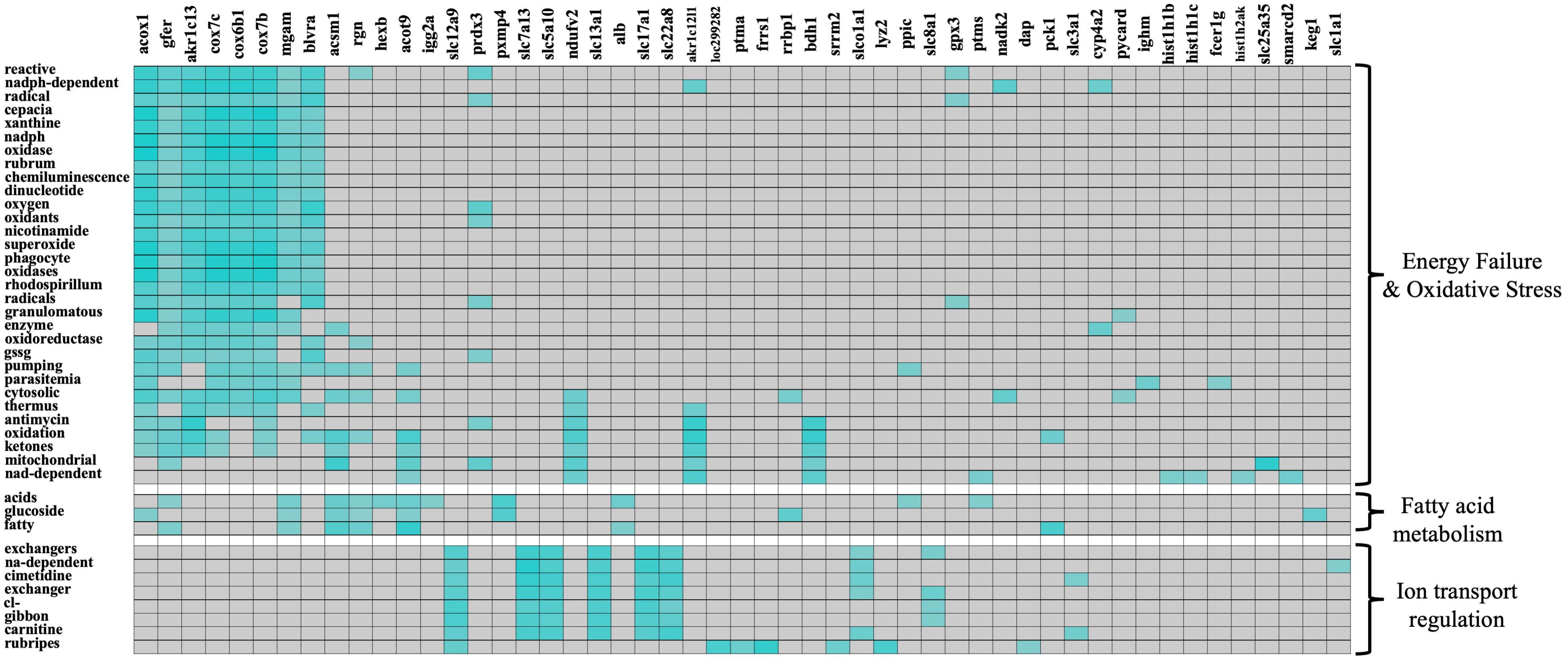

Supplement: Supplementary file 2 — Supplementary Tables. [file 41598_2023_43567_MOESM2_ESM.pdf]
